# Genetic influences on the human oral microbiome

**DOI:** 10.1186/s12864-017-4008-8

**Published:** 2017-08-24

**Authors:** Brittany A. Demmitt, Robin P. Corley, Brooke M. Huibregtse, Matthew C. Keller, John K. Hewitt, Matthew B. McQueen, Rob Knight, Ivy McDermott, Kenneth S. Krauter

**Affiliations:** 1 Department of Molecular, Cellular and Developmental Biology, University of Colorado Boulder, Boulder, CO 80304 USA; 2 Institute for Behavioral Genetics, University of Colorado Boulder, Boulder, CO USA; 3 Department of Integrative Physiology and Institute for Behavioral Genetics, University of Colorado Boulder, Boulder, CO USA; 4 Department of Psychology and Neuroscience, University of Colorado Boulder, Boulder, CO USA; 5 UC San Diego Center for Microbiome Innovation, University of California, San Diego, La Jolla, CA 92093 USA; 6 Department of Pediatrics, University of California, San Diego, La Jolla, CA 92093 USA; 7 Department of Computer Science & Engineering, University of California, San Diego, La Jolla, CA 92093 USA

## Abstract

**Background:**

The human oral microbiome is formed early in development. Its composition is influenced by environmental factors including diet, substance use, oral health, and overall health and disease. The influence of human genes on the composition and stability of the oral microbiome is still poorly understood. We studied both environmental and genetic characteristics on the oral microbiome in a large twin sample as well as in a large cohort of unrelated individuals. We identify several significantly heritable features of the oral microbiome. The heritability persists in twins even when their cohabitation changes. The heritability of these traits correlates with the cumulative genetic contributions of over half a million single nucleotide sequence variants measured in a different population of unrelated individuals. Comparison of same-sex and opposite sex cotwins showed no significant differences. We show that two new loci on chromosomes 7 and 12 are associated with the most heritable traits.

**Results:**

An analysis of 752 twin pairs from the Colorado Twin Registry, shows that the beta-diversity of monozygotic twins is significantly lower than for dizygotic or unrelated individuals. This is independent of cohabitation status. Intraclass correlation coefficients of nearly all taxa examined were higher for MZ than DZ twin pairs. A comparison of individuals sampled over 2-7 years confirmed previous reports that the oral microbiome remains relatively more stable in individuals over that time than to unrelated people. Twin modeling shows that a number of microbiome phenotypes were more than 50% heritable consistent with the hypothesis that human genes influence microbial populations. To identify loci that could influence microbiome phenotypes, we carried out an unbiased GWAS analysis which identified one locus on chromosome 7 near the gene IMMPL2 that reached genome-wide significance after correcting for multiple testing. Another locus on chromosome 12 near the non-coding RNA gene INHBA-AS1 achieved genome-wide significance when analyzed using KGG4 that sums SNP significance across coding genes.

**Discussion:**

Using multiple methods, we have demonstrated that some aspects of the human oral microbiome are heritable and that with a relatively small sample we were able to identify two previously unidentified loci that may be involved.

**Electronic supplementary material:**

The online version of this article (doi:10.1186/s12864-017-4008-8) contains supplementary material, which is available to authorized users.

## Background

Humans support the growth and maintenance of diverse sets of microbes in niches in contact with the environment including skin, lungs, mouth and gut [1]. Studies of these microbes in the gut and oral cavity have uncovered key interactions between bacteria and human hosts in a wide variety of normal and pathological states [2–6]. Many of these interactions are inferred from correlations between the composition of the microbial populations and changes in health status. For example, in gingivitis, an increase in Gram negative and anaerobic bacteria causes inflammation in the mouth [2–6]. Our understanding of the basis for changes in microbial composition, and of how these changes influence human phenotypes, is still a work in progress. Clearly environmental factors and host genetic factors have important influences [3, 4, 6, 7], perhaps best demonstrated to date by studies in the gut [8].

Candidate gene studies have been most effective at identifying human genetic influences on the microbiome. By this approach, informed hypotheses about human genes that may conceivably influence a particular microbiological phenotype (i.e. susceptibility to infection) are tested with family or population-based studies to identify human variants that are statistically consistent with the hypothesis. Examples include MHC genes [9], SLC11A1 [10], the MEFV gene [11], FUT2 gene [12], and loci linked to susceptibility to infectious disease [13]. While often successful, the candidate gene approach is limited by the ability to formulate hypotheses given current knowledge. They are neither comprehensive nor sufficient to identify the entire range of human genes involved in population changes associated with complex phenotypes (i.e. obesity, gum disease) or with maintenance of the composition of the “normal” microbiome. In addition the significant inter-individual variation in microbiome composition often masks specific effects of human genes if insufficient numbers of individuals are studied. Moreover, the microbiome of a niche includes complex mixtures of organisms and is in part defined by interactions among its members making the identification of a “microbial phenotype” complicated.

The oral microbiome is one of the most diverse microbial niches in the human body, including over 600 different microorganisms (Dewhirst et al., 2010). It is in continual contact with the environment, and has been shown to be susceptible to many environmental effects. These environmental factors include tobacco use [14–22], romantic partners [23], and cohabitation [6, 24]. The microbes reside in sub-niches along the oral cavity including on the tongue, cheek, and teeth [1, 25–28]. The salivary microbiome has been shown to be representative of many the oral microbiome niches, which is thought to be due to the fact that microorganisms from the oral cavity surfaces shed into the saliva [28, 29]. Previous salivary microbiome studies have identified specific microbiota that are present in almost all individuals, referred to as the core microbiome [6, 28, 30]. Saliva is also accessible, making it ideal for surveys of populations for microbiome studies.

In this paper, we describe an unbiased approach to studying the effects of human genes on the oral microbiome with a two-step strategy. The first step utilizes twin information to establish heritable phenotypes related to the microbiome; and the second identifies DNA sequence variation associated with the identified highly heritable traits. From 16S rRNA sequence information, a large number of potential phenotypes can be explored with the twin studies to allow identification of the most heritable and therefore the phenotypes most likely to be mapped in the association study. A key strength of this approach lies in the independence of the data underlying the two steps (i.e. MZ/DZ status in the twin study and SNP association data in the second) reducing multiple testing and type 1 effects on the power to carry out the test for association. The ability to refine a phenotype prior to carrying out an association study can lead to greater likelihood of detecting specific SNPs that influence it [3, 4, 31]. We show, with the largest oral microbiome twin study to date, that multiple phenotypes of the salivary microbiome are heritable. Using these phenotypes, we identify promising host gene candidates in a genome wide association study of an separate sample that may play a role in establishing the oral microbiome.

## Methods

### Sample selection and DNA extraction

Twin samples were obtained from the Colorado Twin Registry (Rhea et al. 2006, Rhea et al. 2013). The twin sample included 366 monozygotic pairs (MZ), 263 same sex, and 123 opposite sex dizygotic pairs (DZ). Unrelated individuals were ascertained from community and clinical samples participating in the Colorado Center for Antisocial Drug Dependence and isolation of DNA from saliva and characterization of their genotypes was as previously described [32].

### 16S ribosomal specific PCR and MiSeq sequence determination

Pooled DNA from triplicate PCR with the 16S V4 hypervariable primers 515F/806R was done according to the Earth Microbiome Project 16S rRNA amplicon Protocol, with unique barcode indices for multiplex sequencing on the forward primer [33–35]. Concentration of pooled products was determined by picogreen. 240 ng from each sample was pooled for multiplex paired-end (2X150) sequence determination on the Illumina MiSeq platform.

### Sequencing analysis

The fastx-toolkit (http://hannonlab.cshl.edu/fastx_toolkit/commandline.html) and ea.-utils fastq-mcf package were used to trim and quality filter the forward and reverse reads (https://wiki.rc.ufl.edu/doc/EA-Utils) [36]. The command join_paired_ends.py in QIIME was then used to merge reads using the fastq-join method. Reads with quality score < 25 and that were not between 251 and 254 bps after merging with their paired end were removed. The remainder of the read processing was completed using QIIME v1.9 (Caporaso et al., 2010b). Merged reads were demultiplexed, filtered to remove reads with uncalled bases and barcode mismatches. De novo and reference based chimeras were removed using the USEARCH61 algorithm [37] implemented within QIIME against the gold database (microbiomeutil-r20110519T). Filtered reads were then classified in QIIME against the August 2013 Greengenes 97% reference database. Using closed reference alignment at 97% rather than recently described methods based on high-resolution sequencing methods such as DeBlur [38], DADA2 [39] roth, or MED [40] was used to permit limited but important phylogenetic grouping of likely functionally similar OTUs. Using the higher resolution methods would increase OTU numbers at the cost reducing the numbers of individuals harboring each OTU and thereby reducing power to establish heritability.

### OTU quality filtering

Samples from 1504 twins of whom 111 within-twin longitudinal samples with at least 3500 reads and DNA samples from 1481 unrelated individuals with at least 3000 reads produced 2664 and 2679 OTUs respectively. All samples were rarefied to 2500 sequences to retain as many samples as possible to improve power with little effect to results [41, 42].. To avoid analyses of OTUs that were the result of sequencing or PCR error, OTUs that were not present in at least 2 subjects and observed at least 10 times were removed, resulting in 895 OTUs in the twins and 931 OTUs in the unrelated individuals. One of the unrelated individuals was later removed during analysis due to cryptic relatedness leaving 1480 people in the unrelated sample.

### Beta-diversity analysis

β-diversity was analyzed via Bray Curtis and UniFrac (Unweighted and Weighted) using QIIME (Caporaso et al., 2010b) and R [43]. Analyses included 366 MZ pairs, 386 DZ pairs, and 37,832 unrelated pairs obtained by using age and DNA collection year matched non-cotwin pairs from the twin sets. β-diversity measures between groups were compared via the Wilcoxon-Mann-Whitney test (two tailed wilcox.test in R). *P* values were calculated similarly to as previously described [8]. In short, the pair labels (either MZ, DZ, or unrelated) were permuted 10,000 times and the W test statistic collected from each permutation. The *P* value was then calculated by dividing the number of W test statistics greater than the observed W test statistic plus 1 by the number of permutations plus 1. Biplot analyses were used as implemented in QIIME (Caporaso et al., 2010b). In experiments where cohabitation was required, only cotwins 18 and under and those over 18 who identified themselves as cohabitating were included, which removed 328 subjects from the total twin sample who were living separate from their cotwin. This population of 588 twins pairs is referred to as the “cohabitation sample.” Cohen’s D effect size for β-diversity measurements was calculated using the R package ‘effectsize’ (command ‘cohen.d’) [44].

### Categorization of microbial traits

Microbial traits included taxonomic groups, OTUs, **α** -diversity measurements, and principal coordinates from β-diversity measurements (Additional file 1: Tables S11–14), collapsing all perfectly correlated traits. Microbial traits were then processed within each population separately: twin pairs, European unrelated (EUR), and Admixture American unrelated (ADM). Traits were transformed to z-scores and then categorized as either continuous (at least 85% subjects must have a value >0 and Shapiro Wilk *P* value greater then 1E-28) or categorical (all other traits). Shapiro Wilk test was performed use the R packaged ‘stats’ (command ‘shapiro.test’) [44]. Categorical traits were then binned based upon z-score transformation on all non-zero values (zeros not transformed): zero counts, less than or equal to −1, greater than −1 and equal or less than 0, greater than 0 and less than or equal to 1, greater than 1). Some traits failed to categorize due to lack of variation, resulting in the final trait counts: twins (41 continuous and 955 categorical), EUR unrelated (55 continuous, 945 categorical), ADM unrelated (98 continuous, 807 categorical). Only the continuous traits were used in the EUR and ADM populations so data is provided only for those traits. Descriptions of all traits can be found in Additional file 1: Tables S11–14.

### Intraclass correlation coefficient

The MZ and DZ ICC values were calculated using the R package ‘irr’ (icc command) [44] and were compared using the Wilcoxon Signed Rank Sum test function in the R package ‘stats’ (wilcox.test) [43]. The ICC values were calculated for all taxonomic groups that were categorized to be treated as continuous traits (24 taxonomic groups, Additional file 1: Tables S4 and S11). *P* value was calculated as similarly to as previously described in which the zygosity labels of the twin pairs were randomized 10,000 times and the ICC values then calculated [8]. This analysis compared the overall distribution of the ICC values for the MZ twin pairs compared to the DZ twin pairs. Because the entire distribution was compared and not each taxa individually multiple testing correction was not needed. In addition the ICC values for the remaining 17 continuous traits were determined (Additional file 1: Tables S4 and S11).

### ACE twin modeling

The ACE/ADE univariate twin modeling used the OpenMx package as implemented in R [43, 45, 46] (see Additional file 2: Supplemental Methods). The following covariates were included in the model: age, sex, sequencing run (1–5), and year DNA was collected. The appropriate twin model was selected by analyzing the ratio of 2rDZ to rMZ (if 2rDZ > rMZ use ACE, if 2rDZ < rMZ use ADE). The standardized A was reported as the heritability estimate calculated from the appropriate twin model for each trait (Additional file 1: Tables S5 and S6).

### Host genome genotyping and imputation

Genotypes were obtained as previously described [32]. Ancestry was determined by weighting 43,413 SNPs (MAF > 5%, no AT or GC, low LD) against 1000 Genomes principal components using PCo plots [47]. 469 subjects were identified as Admixture (ADM) and 830 were identified as European (EUR). SNPs were filtered by removing: AT or GC SNPs (107,670), allele switches inferred by the imputation server (1733 SNPs), MAF < 1% (ADM = 25,586, EUR = 76,142), and HWE failure threshold of 0.0001 (ADM = 469, EUR = 25). No filtering based upon missing subject or genotype was needed, because there were no SNPs or subjects with a missing rate greater than 10%. The remaining SNPs (561,204 ADM, 510,818 EUR) were then submitted to the Michigan imputation server using the phase3 reference panel with SHAPEIT for each of the two ancestry groups. The imputation analysis produced 47,072,408 variants for both samples. SNPs with MAF < 1% (based upon dosages), RSQ value <0.8, and multiallelic SNPs were discarded. One ADM and 2 EUR subjects with excessive or limited heterozygosity were removed (heterozygosity ~4 standard deviations from the mean). The imputed SNPs were then pruned for LD with the INDEP function (window size = 50, number of SNPs shift per step = 5, variance inflation factor = 2.0). This LD pruning resulted in deletion of 634,065 SNPs in the ADM population, and 437,921 SNPs in the EUR. These pruned imputed SNPs were then used to calculate the first 10 principal components and the estimated identity by descent (IBD) was used to delete one from each pair of subjects with an IBD > 0.185 (estimated with PLINK v1.9, [48] (number removed ADM = 12 and EUR = 0). In addition subjects that were identified as outliers in the first 10 PCAs were removed (number removed: ADM = 111, EUR = 0). Lastly, analyses were limited to subjects that had no “missingness” for all of the covariates removed in the model (number removed: ADM = 1, EUR = 5). There were then 8,172,048 SNPs to be analyzed in the ADM sample (*n* = 344) and 6,862,363 SNPs in the EUR sample (*n* = 823).

### Genome complex trait analysis

Genome Complex Trait Analysis (GCTA) was performed on all traits categorized as continuous in both the twin and unrelated populations using the GCTA software [49]. The GCTA analysis was performed on the cleaned imputed genotypes described above in the European sample (all IBS estimates <0.025, *n* = 818). The following covariates were included in the model: age; sex; sequencing run (1–5); year DNA was collected; saliva collection method for 16S sequencing; DNA collection method for host genotyping; and the first 10 PCs to control for population stratification. GCTA estimates for the Admixture American sample were not reported due to the small sample size after the threshold of IBS estimates less than 0.025 were applied.

### Genome wide association study

Genome wide association study analyses were performed using the software EPACTS [50]. The Q.EMMAX function was used, analyzing the dosage information for each variant. The GWAS analyses were performed in the ADM and EUR ancestry groups separately. For both analyses a kinship matrix and first 10 principal components were included to control for population stratification within each ancestry sample (described above). In addition to controlling for population stratification the following covariates were included in the model: age; sex; sequencing run (1–5); year DNA was collected; saliva collection method for 16S sequencing; DNA collection method for host genotyping; and tobacco use (for specific analyses). The kinship matrix was created based upon all 22 autosomes using the kinship function in EPACTS. To rule out the possibility that stratification or computational method influenced results, three additional methods utilizing different programs and methods for controlling for population stratification were carried out. These were: EPACTS with only the kinship matrix made from all SNPs (EPACTS kinship); PLINK with the first 10 PCs (PLINK 10 PCs); and GCTA with the leave-one-out kinship matrix (GCTA kinship loco). For all methods the following covariates were included in the model: age; sex; sequencing run (1–5); year DNA was collected; saliva collection method for 16S sequencing; and DNA collection method for host genotyping.

### Genome wide association study meta-analysis across ancestry

ADM and EUR GWAS analyses were combined in a meta-study using the METAL package. METAL analyses were performed on overlapping SNPs with the “samplesize” scheme in which the *P* value and direction of effect for each variant is weighted by sample size correcting the test statistics for population stratification with the “genomiccontrol” option. The results of the METAL analysis were then re-run through the program to confirm that population stratification was properly controlled for as suggested by the METAL guidelines. QQ-plots were created in R using the package “qq-man” [43, 51].

### Data access

The 16S rRNA gene sequencing data from this study has been submitted to the EMBL-EBI under study numbers ERP023086, ERP023087, ERP023088, ERP023089, ERP023090, and ERP023091. The host genome sequencing data used in this study was made publically available by Derringer et al. 2015.

## Results

### Twin analysis of the host genetic contribution to microbiome composition

We performed an analysis of 752 twin pairs from the Colorado Twin Registry [52, 53] to estimate host genetic and environmental contributions to salivary microbiome composition. The sample included 366 monozygotic pairs (MZ), 263 same sex, and 123 opposite sex dizygotic pairs (DZ) that ranged from 11 to 24 years of age. Taxonomic analyses using sequencing of variable region IV of the 16S rRNA amplicon prepared from the saliva of each twin was carried out using QIIME [54] on high-quality Illumina MiSeq paired end reads as previously reported [8, 54]. We determined phyla abundances to be Firmicutes (56%), Proteobacteria (13%), Bacteriodites (13%), Actinobacteria (12%), and Fusobacteria (6%) from the 2664 operational taxonomic units (OTUs) found, which is consistent with the “core” salivary microbiome we and others have previously reported [1, 6, 25–28, 55]. All of our analyses included only OTUs that were present in at least 2 subjects and observed at least 10 times in total after rarefying at 2500 reads. This filtering yielded 895 OTUs that were considered for all subsequent experiments.

**Fig. 1:**
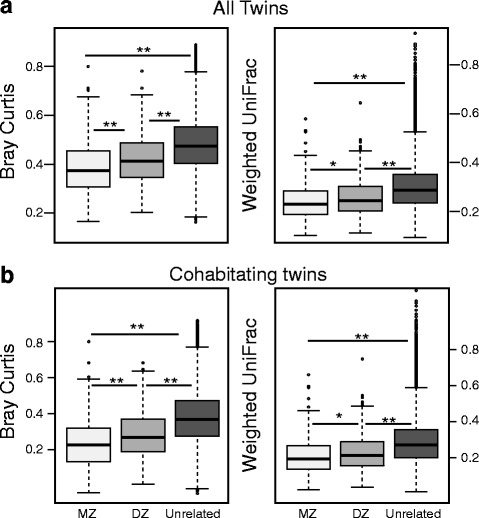
Beta diversity comparisons between twin pairs: Beta diversity measurements for MZ(*n* = 366 pairs), DZ(*n* = 386 pairs, and unrelated individuals(*n* = 37,832 pairs). Mann-Whitney U two-tailed test was applied between groups (Bray-Curtis and Weighted UniFrac Beta Diversity Measurements shown). *P*-values were determined with 10,000 permutations of the group labels. **a**. Entire twin sample(*n* = 752 pairs) **b**. only twin pairs either 18 and younger or those over the age of 18 and living together(*n* = 588 pairs). (**) *p*-value < 0.0005, (*) *p*-value < 0.05)

Measurements comparing mean β-diversity among MZ, DZ and unrelated individuals allows for assessment of microbial population differences between groups. With either Bray-Curtis [56] or Weighted UniFrac [57, 58] measures of β-diversity among MZ twin pairs were significantly more similar to each other than DZ twin pairs, and for all 3 β-diversity measurements (Bray-Curtis, Unweighted and Weighted Unifrac) MZ and DZ twin pairs were significantly more similar to each other than to unrelated individuals (see Fig. 1a). This analysis was also carried out with abundant OTUs (i.e. present in at least 50% of the subjects) and all OTUs (i.e. no filtering or rarefaction) with very similar results (Additional file 2: Figures S1 and S2). Rarefaction at 2500 reads produced consistent results across all rarefactions (Additional file 2: Figure S5), so for subsequent analyses, one rarefaction to 2500 reads is shown. We could detect no significant effect on any β-diversity measure due to sex when comparing same sex vs opposite sex dizygotic twin pairs perhaps because the sample size did provide enough power to differentiate sex effects from inter-individual variation (see Additional file 2: Figure S6). In subsequent DZ analyses therefore, opposite sex pairs were included.

The Colorado Twin Registry [52, 53] includes highly detailed phenotypic information that is invaluable in identifying and controlling for environmental confounders that may play an important role. Living together is a covariate influencing microbial populations in humans [6, 24]. It is well-known that MZs tend to cohabitate longer than DZs [59, 60] and indeed our previous work has shown that shared environment influences the oral microbiome [6]. Therefore, it was possible that the tendency of MZ cotwins to live together longer could be driving the observed heritability. To examine this potential confounder, we reanalyzed the data in Fig. 1a based on questionnaire data from the sample in which we restricted the analysis to only cohabitating pairs (i.e. 18 years age or younger and removal of cotwins reporting living apart). While ideally we would have also analyzed only twin pairs living apart, our sample size did not permit it. As seen in Fig. 1b, MZs remained significantly more similar to each other than DZ twin pairs for the Bray-Curtis and Weighted UniFrac measurement, and was also observed in the abundant and unfiltered/unrarefied OTU tables described above (Additional file 2: Figures S3 and S4). We conclude that cohabitation does not play a significant role in the observed microbiome heritability.

To quantify the differences between groups the Cohen’s D effect size was calculated for all β-diversity measurements (Additional file 1: Tables S1 and S2) for both the full sample and the sample limited to twin pairs who were cohabitating (Cohen 1992). Comparisons between the unrelated and twin pairs yielded medium to large effect sizes. All other comparisons were either small or negligible, the largest of which being between MZ and DZ pairs for Bray Curtis. To quantify the effect cohabitation had on β-diversity measurements the effect size between all twin pairs (either MZ or DZ) and just pairs living together (either MZ or DZ) were compared for all measurements yielding only negligible effect sizes (Additional file 1: Table 3) consistent with a conclusion that cohabitation was not driving observed heritability.

The stability of the oral microbiome over time in adults is reported to be remarkably high relative to that of other body sites [1, 30, 55, 61]. To confirm and extend this observation, we assessed the stability of the oral microbiome in longitudinal samples from our cohort for 111 individuals, 2–7 years apart (mean = 5 yrs). The mean β-diversity measurements between longitudinal samples were compared to the mean of unrelated individuals of different ages. For all three β-diversity measurements examined (Bray Curtis, Unweighted and Weighted UniFrac) subjects were significantly more similar to themselves than were unrelated individuals (Additional file 2: Figure S7).

**Fig. 2:**
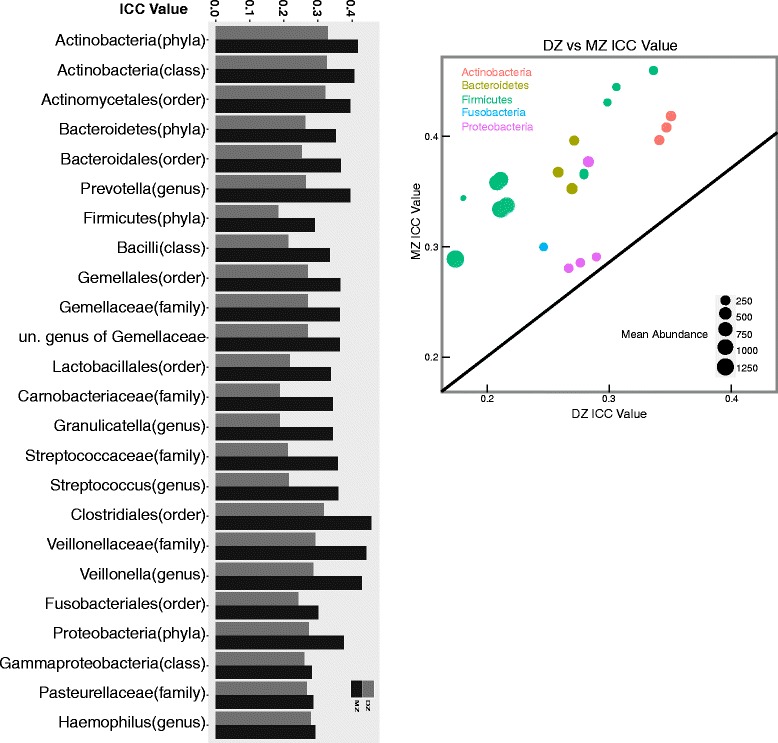
Intraclass correlation coefficient comparison: Intraclass correlation coefficient values for MZ and DZ twin pairs of taxa abundances(*n* = 752 pairs). Taxa are grouped by phyla in the bar graph. The ICC values between MZ and DZ twin pairs were compared with the Wilcoxon Signed Rank Test with 10,000 permutations of the zygosity labels (Pvalue==0.00629937). The inset shows a plot of the ICC values for MZ vs. DZ (line is where MZ = DZ)

Intraclass correlation coefficients (ICCs) are useful for estimating heritability of individual observations within a group of related observations (i.e. the abundance of specific salivary taxa between MZ pairs); the higher the ICC values for MZ pairs compared to DZ pairs, the greater the heritability [62]. As shown in Fig. 2, ICC values for essentially all abundant taxa are significantly greater in MZ than DZ pairs. No significant difference was observed between the same sex and opposite sex DZ pairs across the taxa analyzed (Additional file 2: Figure S8) [8]. The set of taxa analyzed were those that were categorized as continuous (see Methods). Significance was established with Wilcoxon Signed Rank tests strongly supporting the heritability of taxon abundance in this twin set. We also tested 4 different alpha diversity measures (Shannon Index, Chao1, Observed OTUs, PD-Whole Tree), the first 3 principal coordinates (PCo) for three different β-diversity measurements (Bray Curtis, Unweighted and Weighted UniFrac) and saw that most traits were consistent with the conclusion that MZ cotwins are more similar than DZ cotwins. A complete list of the 41 phenotypes tested and their ICC values can be found in Additional file 1: Tables S4 and S11.

### ACE modeling identifies heritable microbiome phenotypes

Twin modeling approaches are used to estimate the amount of variance attributable to additive genetics (A), common environment (C) or dominance (D), and unique environment (E) [46]. An ACE or ADE model was constructed for each of 946 traits including alpha diversity, principal coordinates (PCos) of β-diversity of taxonomic groups, and individual OTUs. A complete list of the A, C/D, and E values for each of these phenotypes can be found in Additional file 1: Table S5. A power analysis shows that our sample is well powered to model continuous traits but is underpowered for categorical traits (Additional file 2: Figure S9). Traits that were not categorized as continuous were treated as categorical traits (see Methods). Therefore, while still of interest, the categorical traits should be viewed with lower confidence (see Additional file 2: Supplemental Methods). In the twin models both C and D cannot be modeled at the same time since each captures the same variance, but the genetic contribution (A) can be compared between phenotypes modeled with ACE or ADE models.

Of the 946 traits 55% were modeled as ACE and 44% ADE. Averaging heritability estimates (A) for traits within each phenotype category described above (i.E. *alpha* diversity, β-diversity PCos, OTUs, taxa) a trend that PCos of measurements have the highest mean heritability estimates emerged for either the full sample or to just twin pairs that are cohabitating (Additional file 2: Figure S10). The most heritable were OTU4483015 that corresponds to an unnamed species of *Granulicatella* (55.8% heritable, 95% CI: 0.282–0.634, corrected *P* value 0.0405) and PCo 2 for Bray-Curtis (46.3% heritable, 95% CI: 0.233–0.551, corrected *P* value 0.0405) (see Additional file 1: Table S5). To better understand which taxa were driving this PCo a QIIME biplot analysis [54] identified the genus Streptococcus as the most abundant taxon on the first 3 principal coordinates from Bray-Curtis (Additional file 2: Figure S12). Repeating the ACE models excluding twin pairs who reported that they had moved out after age 18 (i.e. modifying effects of C and/or E in the model) did not greatly alter the heritability estimates or other components of the model (Additional file 1: Table S6, Additional file 2: Figures. S10 and S11). The unique environment (E) accounted for most of the variation of the traits tested in both the full and cohabitation sample (Additional file 2: Figure S11). Little change in the common environment (C) was observed between the full and cohabitation sample analyses (Additional file 2: Figure S11).

We compared phenotypes deemed to be heritable in our study (44 traits with Benjamin-Hochberg corrected *P* values of less than 1) with phenotypes seen to be heritable in 5 studies of gut [63–67] and 1 in dental plaque, [68]. We found that 14 of the 44 traits were mentioned with heritability estimates of at least 1% in one or another study, though none showed high statistical significance (Additional file 1: Table S16, Additional file 2: Supplemental Methods). This is consistent with the possibility that genes that may drive the heritability in the salivary microbiome may also have more general influences in other human niches.

### SNPs correlate with observed heritability

It is assumed that host genes interacting with the oral microbiome are responsible for the observed heritability. The best way to identify them is by the analysis of an association between genetic variation (i.e. SNPs) and traits. The power to detect this is a function of the number of individuals, the number of tests and the number and types of SNPs available. The greatest power to uncover association given a fixed sample size is obtained by analyzing a limited number of phenotypes (hypotheses) based on prior information rather than repeatedly testing multiple hypotheses on the same data [69]. To limit hypotheses to test we focused on the traits found most heritable in twin studies. Traits found to be most heritable are expected to produce the best results in a genome-wide association (GWAS) study.

DNA was previously prepared from saliva and blood of 1480 individuals unrelated to the twins and to each other [32]. Human DNA from this sample was subjected to Affymetrix Chip-based genotype analysis that resulted in 696,388 validated human SNP genotypes per individual [32]. The age of subjects ranged from 11 to 33 years and 29% were female. Ancestry was assigned by weighting a subset of the genotyped SNPs against the 1000 genomes dataset and assigning individuals to ancestry group using principal coordinate analysis plots [47]. The genotyped SNPs were then quality filtered and submitted to the Michigan Imputation Server (https://imputationserver.sph.umich.edu/index.html#!run/) for phasing and imputation (see Methods). After quality filtering this produced 6,862,363 European (EUR) and 8,172,048 American Admixed (ADM) imputed variants respectively that were used in all subsequent analyses. Imputed SNPs from two different randomly selected chromosomal areas in 68 individuals were resequenced with Sanger sequencing to validate imputation. We found that 65/68 imputed calls validated completely with 3 apparently incorrectly imputed (data not shown). We conclude that imputation provides significantly greater resolution to SNP-based maps at little cost to accuracy.

**Fig. 3:**
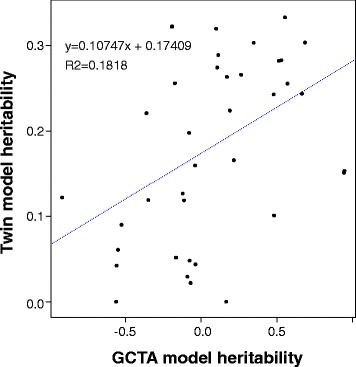
Heritabiltiy estimate comparison: Twin model heritability estimates vs amount of variate accounted for by common single nucleotide (“heritability”) via GCTA for the Euoropean population (*n* = 818) for traits continuous in both samples (*n* = 40). The correlation between the estimates is significant with a *P* value of 0.00609

The salivary microbiome of the 1480 individuals was characterized by 16S RNA sequencing identifying 2679 OTUs, where again as in the twin study, the most prevalent phyla were Firmicutes (55%), Proteobacteria (14%), Bacteriodetes (14%), Actinobacteria (11%), and Fusobacteria (6%). Filtering by prevalence and abundance as described above produced a total of 931 OTUs used for our studies. The SNP-based heritability of microbiome phenotypes in the unrelated population was assessed using Genome Complex Trait Analysis (GCTA) [49] that estimates the amount of phenotypic variance that can be explained by SNP-based composite genetic variance. To avoid false positives, the genetic relationship matrix was limited to subjects that were estimated to have IBD < 0.025. The first 10 ancestry principal components from LD-pruned (linkage disequilibrium) SNPs were included to control for population stratification (see methods). Given the relatively small sample size, single trait heritability estimates were not evaluated but rather gross trends were observed across all continuous traits. A positive correlation was observed between the heritability estimates from AC/DE twin models and the European GCTA analyses (Fig. 3) with a disattenuated correlation of 0.831 (Additional file 2: Supplemental Methods). The mean heritability estimates across all continuous traits in the European sample was 0.0563 (SE = 0.371, *n* = 55 traits). OTU4446902 (unnamed species of the family Gemellaceae) and its corresponding taxa levels (order, family, and genus) showed suggestive significant GCTA heritability estimates after controlling for multiple testing (OTU4446902 V(G)/Vp = 0.944 SE = 0.357 *P* value-BH corrected = 0.053, see Additional file 1: Table S15, Additional file 2: Supplemental Methods). However, these traits were not observed to be heritable in the twin models (Additional file 1: Tables S11, S13, S15). The small sample size was not expected to result in significant GCTA *P* values although it has been noted that the meaning of such *P* values is limited but even in small samples observable trends can be meaningful [70]. Nevertheless, it is striking that both twin studies and GCTA on separate samples show heritability across the same continuous traits (Fig. 3). This is consistent with the expectation that genome sequence variation is a basis of observed heritability.

### Genome wide association study with heritable phenotypes

We ranked the continuous traits based on their heritability (the top trait showing a significant Benjamin-Hochberg corrected *P* value of 0.0405 shown in Additional file 1: Table S5) and performed a genome-wide association of the top six with the *E*fficient and *P*arallelizable *A*ssociation *C*ontainer *T*oolbox (EPACTS) [50]. This would be expected to reduce the loss of power due to multiple testing of hundreds of phenotypes. The family Carnobacteriacea was excluded from the GWAS analyses since it was highly correlated with the genus *Granulicatella* (R^2^ = 1) and the latter has a more refined taxonomic resolution. It is well established that continuous traits afford greater power in both twin studies and in GWAS [71, 72]. Therefore, although some categorical phenotypes (i.e. not observed to be continuously distributed) showed high twin heritability (see Additional file 1: Table S5), for GWAS we only studied continuous traits. The analyses were all controlled for age, sex, and sequencing run among other covariates (see methods). Analysis was done independently with individuals from the two major different ancestry groups of the unrelated sample, European (*n* = 823) and Admixture (*n* = 344) [71]. Due to the limited size of the admixture sample, only the European sample is discussed and the admixture was only considered for the meta-GWAS discussed below.

**Fig. 4:**
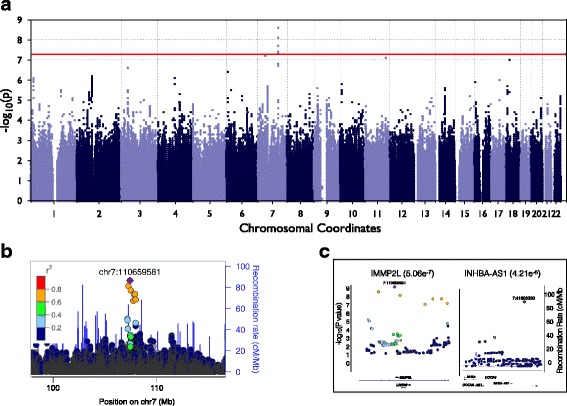
GWAS of genus *Granulicatella*. **a** Manhattan plot of the GWAS analysis in the European ancestry sample(*n* = 823). The *red line* represents the threshold of genome wide significance(*p*-value < 5 × 10^−8^). The abundance of the genus *Granulicatella* was transformed to z-scores in R and used as the phenotype for the European GWAS (see Methods). **b** Locus Zoom plot of the chromosome 7 at the most significant GWAS hit. **c** SNP plot for the genes IMMP2L, INHBA-AS1, and full coding gene of INHBA of the KGG analyses of the GWAS for the abundance of the genus *Granulicatella*

To control for population stratification a kinship matrix created from all the chromosomes and the first ten principal components from the LD-pruned SNPs were included as covariates (see methods). To control for the fact that 6 traits were tested, the genome wide significance level was lowered to 8.33e-09 (5e-08/6traits) (Additional file 2: Figures. S13 and S14). Using this threshold, we found that the genus Granulicatella was significantly associated with the SNP chr7:110,659,581 (*P* value = 2.251e-09, Fig. 4a, QQ Plot Additional file 1: Table S7, Additional file 2: Figure S14) within an intron of the IMMP2L gene on chromosome 7. This gene is known to be involved in mitochondrial protein trafficking [73–75]. The regional Manhattan Plots in Fig. 4b show that the peak locus includes SNPs of decreasing r^2^ values around the peak SNP lending greater confidence to the association. Without a replication sample this result is provisional but potentially interesting. Using PLINK 1.9 [48], which takes categorical imputed genotypes rather than the probabilistic dosage calls produced by imputation as input, produced results consistent with this association (data not shown) showing the association is independent of underlying computational method.

A comparison of the 100 SNPs with lowest *P* values in each of the six phenotypes examined in the European sample revealed that 7 SNPs were held in common between at least two of the phenotypes. Bray Curtis PCo2, Unweighted UniFrac PCo2, and Weighted PCo2, all β-diversity measures, were most often shared (comparisons not shown). After the initial analyses of the 6 most heritable traits, a GWAS was completed in the remaining 64 continuous traits in the European sample. No variant was found to be significant after controlling for multiple testing for these additional tests ((5e-8)/70 = 7.142857e-10) (data not shown).

We have used a relatively conservative approach to controlling for population stratification (kinship matrix + first 10 PCs). To evaluate if this may have produced false negatives, we repeated the GWAS with EPACTS kinship only, PLINK 10 PCs, and GCTA LOCO (leave one chromosome out) (see Methods). Each consistently identified the same SNP at chr7:110,659,581 significantly associated with the trait along with nearby SNPs in high LD associated as well (Additional file 1: Table S17). No additional significant SNPs were identified consistent with the hypothesis that stratification methodology had little effect on identifying the top SNPs and that we were not “overfiltering” with rigorous kinship controls. For completeness, we then carried out a GWAS analyses for the remaining 64 continuous microbial phenotypes using the EPACTS kinship only analyses adjusting significance for the additional multiple testing and found no SNPs to be significantly associated. This is perhaps not surprising given the relatively small sample size (data not shown).

### Meta- and gene-based GWAS analyses

**Fig. 5:**
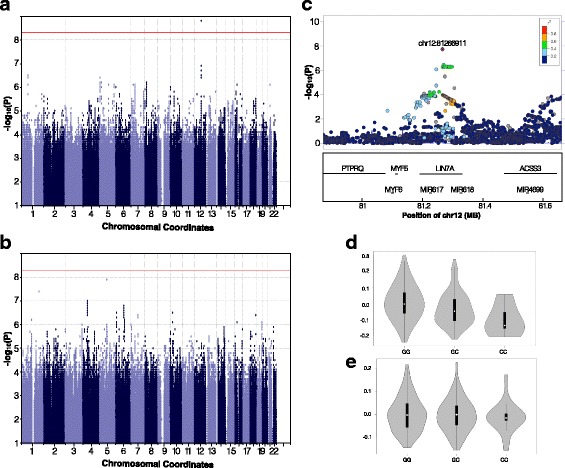
GWAS of Unweighted UniFrac principal coordinate 3. Principal Coordinate 3 of Unweighted UniFrac was transformed to z-scores in R and used as the phenotype for the GWAS analyses. **a** Manhattan plot of the GWAS analysis in the European ancestry sample(*n* = 823) and **b** Admixture American ancestry sample (*n* = 344). **c** Locus Zoom plot of the chromosome 12 at the most significant Meta-GWAS hit. **d**-**e**. Violin plots of the Principal Coordinate 3 of Unweighted UniFrac for each genotype within each ancestry population (**d** EUR: GG *n* = 705, GC *n* = 112, CC *n* = 6; **e**. ADM: GG *n* = 193, GC *n* = 133, CC *n* = 18)

The size of the ADM sample made it unlikely to produce statistically significant results. To glean useful information from it we combined it with the EUR data described using a meta-analysis approach that can effectively deal with population issues inherent in mixing samples of different populations. METAL [76] is such a meta-analysis package that takes as input individual SNP *P* values and the direction of their effects weighted by the sample size to arrive at composite *P* values. The test statistics were also corrected for population stratification (see methods). The METAL analysis identified the same suggestive significant SNP on chromosome 7 that was associated with *Granulicatella* abundance in the EUR GWAS (chr7:110,659,581, *P* value = 2.51–09, see Additional file 1: Table S8 for complete results). However, due to the small size of the ADM sample, this SNP did not survive quality filtering in the METAL analysis and so was not a factor in the METAL analysis outcome. Analyses of Unweighted Principal Coordinate 3 yielded a SNP on chromosome 12 that reached genome wide significance in the same direction (positive beta) for the combined sample, though it was not robust to multiple testing correction (chr12:82,166,911, *P* value = 1.845–08, Fig. 5a–b, Additional file 1: Table S9). Again, the regional Manhattan Plots in Fig. 5c show the peak locus includes SNPs of decreasing r^2^ around the peak SNP consistent with the association. The minor allele C, was shown to be consistent with lower PCo3 z-scored values (Fig. 5d–e).

The most promising single SNP association occurred with the phenotype defined as the abundance of the genus *Granulicatella*. We reanalyzed the association data with the gene-based tool Knowledge-based mining system for Genome-Wide Genetic studies (KGG4, [77] that constructs whole gene association scores from a summation of SNP *P* values contained in each gene. The abundance of *Granulicatella* identified two genes on chromosome 7 as highly associated: a protein coding gene IMMP2L (corrected *P* value = 0.0176) involved in protein processing associated with mitochondrial import and a non-coding antisense RNA INHBA- AS1 (corrected *P* value = 0.0488) (Fig. 4c, see Additional file 2: Supplemental Methods). A SNP in INHBA-AS1 had been previously identified in a dental caries GWAS along with a loci in the INHBA gene [78]. INHBA is thought to be important to tooth development, which could have potential interesting implications to the oral microbiome [78–83]. The meta-GWAS results on the PCo3 of Unweighted UniFrac most highly associated region was the gene LIN7A on chromosome 12 (corrected *P* value = 0.2107, see Additional file 2: Supplemental Methods).

### A strong environmental covariate fails to influence top-scoring associations

Tobacco use correlates with changes in the oral microbiome and the abundance of specific taxa [14–18, 20–22]. It was possible that tobacco or other factors influenced our observation of genetic association. For example, *Streptococcus* abundance, a highly heritable phenotype, has also been shown to change in smokers [14, 16, 17, 20–22, 84]. In addition other substances could potentially change the oral microbiome. Among these alcohol [20] and marijuana, though these effects have yet to be determined. However, marijuana use is correlated with poor oral health, which is often indicative of changes in the oral microbiota [85–88]. We had available the self-reported tobacco, alcohol and marijuana use in 92% of our subjects for the previous six months. We therefore repeated the analyses using the three substances as covariates (see Additional file 2: Supplemental Methods). As seen in Additional file 2: Figures. S15 and S16, controlling for tobacco/alcohol/marijuana use had negligible impact on the top hit on chromosome 7 for the genus *Granulicatella* (see also QQ plots, Additional file 1: Table S10). For the 6 highly heritable continuous traits that were analyzed, both with and without substance use covariates, results appear to be consistent with and without substance (Additional file 2: Figures S15 and S16).

## Discussion

We have shown that microbe abundance and some aspects of the microbial population structure are influenced by heritable traits in saliva. We have ranked the “most heritable” traits using ACE/ADE modeling and GCTA-based SNP heritability and carried out an unbiased GWAS on the 6 most heritable traits. One SNP on chromosome 7 in the gene IMMPL2 reached genome-wide significance. Another gene IINHBA-AS1 on chromosome 7 achieved genome-wide significance when analyzed by KGG4 that relies on a composite association score including all SNPs in each known gene. The significance of these associations was not influenced by “p-hacking” statistical biases common in GWAS because phenotype choice was not based on previous association tests. This approach is a model for using heritability to reduce the multiple testing problems seen in many GWAS reports and it could be the method of choice in the design of GWAS studies in which sample size may be limited.

Bray-Curtis, Weighted UniFrac, and to a lesser extent Unweighted UniFrac β-diversity demonstrate that many components of the microbiome community are heritable (Fig. 1). While a shared environment and behavioral habits contribute to a more similar microbiome (i.e. individuals living together have more similar microbial populations [6, 24]), such studies did not control well for the clear genetic influences in their populations. When we examined the differences among MZ and DZ cotwins and age-matched unrelated individuals that we were confident cohabitated (i.e. removed those who did not live together), the genetic influences remain clear. It is significant that the genetic effects are detected using measures that include all detectable OTUs. To assess heritable influences of individual microbial components, we carried out intraclass correlation analyses that show that heritability extends across nearly all observed taxa individually (see Fig. 2). The one exception is in the fusobacteria where ICC does not distinguish MZ and DZ. Possibly these organisms, known to be “bridges” between early and late colonizers on gum and tooth surfaces [89, 90], may not have interaction with host proteins and could lack human genetic influences.

GWAS of complex traits on relatively small samples is problematic due to the lack of statistical power. The influence of individual genes on traits that have multiple genetic components may be small. Moreover, the microbiome is a highly complex population with interacting networks of bacteria that all may have multiple interactions with the host. A variety of covarying network modeling approaches have demonstrated how complex these communities are [91]. It has been shown that assuming the number of causal variants and their frequency spectra for a pair of traits are similar, more heritable traits are more likely to be detectable in GWAS [31]. Therefore we focused on those microbiome endophenotypes with greatest additive genetic heritability for GWAS. Both ACE/ADE modeling and GCTA SNP heritability are suited to this approach.

The microbial phenotypes with greatest additive genetic influence in the ACE/ADE model on the entire twin cohort were the abundance of the OTU4483015 that corresponds to an unnamed species of *Granulicatella* (twintrait521, heritability 55.8%, 95% CI: 0.282–0.634) and PCo2 of Bray Curtis (twintrait1022, heritability 46.3%, 95% CI: 0.233–0.551, Additional file 1: Table S5). The influence of additive genetics was variable depending on the trait when comparing the full sample to heritability only among cotwins that cohabitate (i.e. under 18 or 19 and older and reported living at home (Additional file 1: Table S6, Additional file 2: Figures S10 and S11). The variation in estimates may reflect environmental effects or loss of power between the full sample (*n* = 752 twin pairs) and the cohabitating sample (*n* = 588 twin pairs) (Additional file 2: Figure S9. Nevertheless, cohabitation did not remove the significant genetic influences. In comparing those OTUs identified as heritable in saliva to those identified in recently reported studies in the gut, we found no obvious overlap (Additional file 1: Table S16). This again points to the complex nature of the microbe-host interactions in primarily aerobic and anaerobic environments and how human genetic influences must also be complex.

As a further test of heritability prior to GWAS, we examined SNP-based heritability in our unrelated sample with GCTA. A positive correlation was observed between the ACE/ADE and GCTA ‘heritability’ estimates for continuous traits in both the full twin sample and the EUR sample (Fig. 3). Previous studies have demonstrated that large samples are needed to produce results reaching statistical significance using GCTA. In their original paper Yang et al. showed that while increasing the sample size does decrease the error bars of the heritability estimates, the heritability estimates themselves remain relatively stable. While the GCTA estimate was not significant upon correction for multiple testing, the positive correlation between the unrelated individuals and the twin studies (0.1818) provides support for the conclusion that for these continuous traits genetic variation influences microbial populations.

A GWAS analysis with the six most heritable continuous traits determined from the twin modeling was carried out in the European (EUR) populations (defined above). The GWAS of the abundance of the genus *Granulicatella* identified a genome wide significant SNP on chr7 (chr7:110,659,581, *P* value = 2.51–09). This SNP is located in an intron of the IMMP2L gene. The GWAS meta-analyses combining the EUR and ADM samples using METAL with the same 6 traits showed no new information about the chr7 SNP due to its low frequency in the ADM population but did produce an additional association with suggestive significance, chr12:82,166,911 (*P* value = 1.845–08) for the phenotype Unweighted UniFrac PCo3, though it was not robust to correction for multiple testing. This SNP is located in the gene LIN7A that is widely expressed in endothelial cells. Markers in LD with the top SNPs (i.e. high r^2^) were also highly associated with the phenotype, but in addition, markers of somewhat lower LD (i.e. low r^2^) that were nearby also displayed elevated significance for both hits. This provides an argument that these loci may not be due purely to chance (Figs. 4b and 5c).

To be adequately powered one must have a large sample size or the single SNP effect must be very large. However, most complex traits are polygenetic and so many loci with small effects account for the variation of the trait. Therefore, where sample size is limited, it may be difficult to observe significant SNP associations. To address this, it is possible to use biological information to inform analyses and increase statistical power. This may be done by aggregating the association of multiple SNPs known to be present within a known gene. By this approach, the possibly small effects of all SNPs in the gene are combined and then the association of the entire gene may be determined. Even if no single SNP is found to be genome-wide significant the combined SNP contributions across the gene may be. One widely used gene-based GWAS analysis method is the Knowledge-based mining system for Genome-wide Genetic Studies (KGG4) [77, 92–95].

An analysis by KGG4 confirmed the gene IMMP2L (corrected *P* value = 0.0176) and additionally identified a non-coding RNA INHBA- AS1 (corrected *P* value = 0.0488) (see methods) as significant hits. IMMP2L functions in the mitochondrion where it is involved with processing of signal peptides as a peptidase directing transport to the interior mitochondrial space [73–75]. INHBA- AS1 and INHBA (closely linked) were previously associated with dental caries in a GWAS, and INHBA was postulated to influence the development of dental caries via its role in tooth morphology development [78]. In support of this hypothesis Zeng et al. discuss that INHBA has been shown to be important for tooth development and knockout mice of INHBA have alterations in the eruption of new teeth [78–83]. Attachment to the tooth surface is a part of the establishment of the oral microbiome and disruption of this process could lead to changes in the community structure of oral biofilms. Ascribing functional significance to IMMP2L, INHBA-AS1, or LIN7A, is speculative in the absence of a replication experiment. Nevertheless, this study is among the first to use heritability to refine microbiome phenotypes prior to GWAS testing and the findings will provide a basis for additional genetic studies in larger replication samples and in future molecular analyses.

Of the 100 most significantly associated SNPs for each of the 6 GWAS analyses in the EUR sample, 7 SNPs were shared at least twice among Bray Curtis PCo2, Unweighted UniFrac PCo2, and Weighted UniFrac PCo2 analyses probably due to shared underlying variation of PCo2. A comparison of SNPS from the Granulicatella GWAS and the PCo3 unweighted UniFrac Meta-Analysis in our experiments with other published GWAS studies of the microbiome found that the majority of overlapping SNPs followed a normal distribution, and those few that did deviate from expectation did not reach genome wide significance in either study (see QQ-plot, Additional file 2: Figure S18) [3, 64–67]. It is perhaps not surprising that genes showing influence in gut do not appear in salivary samples. There is very little overlap in organism composition between niches and it can be argued that one reason for this is that different genes influence each niche.

Genes and environment potentially contribute to all aspects of the microbiome. Whereas twin studies are particularly powerful in differentiating between them, GWAS is poorly suited to teasing these factors apart. We show that tobacco/marijuana/alcohol use has little influence on the ability to detect associations of our top scoring loci. This is somewhat unexpected in that it is well known that some microbes either increase or decrease in response to tobacco [14, 16, 17, 19–22]. This is consistent with a hypothesis that the tobacco effects seen (for example increases in streptococcus abundance) are mostly free of significant genetic influences and that conversely, the genetic effects we find do not dependent on environmental perturbations to be observed. The results point out a need for well-controlled gene by environment experiments to fully understand how genes work and how environmental factors actually influence microbial communities.

## Conclusions

In this study we have shown, using the largest twin oral microbiome study to date, that the oral microbiome is heritable. While cohabitation is clearly a factor in microbiome similarity between co-twins, the genetic effects are observable independent of cohabitation. Twin modeling and correlation of twin models with additive SNP heritability in unrelated individuals determined by GCTA confirmed that observed heritability is the result of genome sequence variation. Prioritization of the most heritable microbial phenotypes reduced the multiple testing problems inherent in some GWAS analyses and allowed us to carry out a successful GWAS analysis of 6 microbiome phenotypes. Future work will focus on replicating these studies in a large independent sample but on its own, it demonstrates that at least some aspects of oral commensal populations are determined by host genetic factors.

## Additional files

Additional file 1: Supplemental_Tables. (XLSX 577 kb)

Additional file 2: Supplemental_Information. (PDF 28601 kb)
