# Supplementary material for: Genetic influences on the human oral microbiome
Source: BMC Genomics. 2017 Aug 24;18:659. doi: 10.1186/s12864-017-4008-8 (PMC5571580; doi:10.1186/s12864-017-4008-8)
Supplement: Supplementary file 2 — Supplemental_Information. (PDF 28601 kb) [file 12864_2017_4008_MOESM2_ESM.pdf]

Supplemental Information

Supplementary Figures

Figure S1. Abundant OTU table beta diversity comparisons …..……………………………..…..3

Figure S2. Unrarefied and unfiltered OTU table beta diversity comparisons………………..……4

Figure S3. Abundant OTU tables beta diversity comparisons limited to cohabitating twin pairs……………………………………………………………………………………………..5

Figure S4. Unrarefied and unfiltered OTU tables beta diversity comparisons limited to cohabitating twin pairs………………………………………………………………………………6

Figure S5. Rarefaction comparison ……………………….………………..………………….………7

Figure S6. Dizygotic opposite sex vs same sex pairs comparison across rarefactions..………..8

Figure S7. Longitudinal samples …………………….……….……….……….……..…………..9

Figure S8. Dizygotic opposite sex vs same sex pairs comparison with ICC ………………..……10

Figure S9. Twin model power analysis …………………….…………….……….…………….…..11

Figure S10. Twin model average heritability estimates ….……….……………….………….……12

Figure S11. Twin model pie charts …..……………….………………………….………………..…..13

Figure S12. Biplot analysis ……………………….…………….…………….……………….….…14

Figure S13. Manhattan plots of European GWAS….……….………….……..……………..…….15

Figure S14. QQ plots of the European GWAS………….…….……….…….………………..….16

Figure S15. Manhattan plots of European GWAS analysis with tobacco/marijuana/alcohol use as covariate……..………….…..………………………………………………..…………..17

Figure S16. QQ plots of European GWAS analysis with tobacco/marijuana/alcohol use as covariate...……………. ……….…………………………...………………………………..18

Figure S17. Ancestry principal components…….………………………………………….…………19

Figures S18. QQ plots of Comparison SNPs..20

Supplemental Methods...21-22

References...23

Supplementary Tables

All tables are stored in the Supplemental Excel sheet on separate tabs labeled ST1-16

Table S1. Cohen's D effect size for full sample..ST1

Table S2. Cohen's D effect size limited to cohabitating twin pairs ST2

Table S3. Cohen's D effect size full sample vs cohabitating twin pair........................ST3

Table S4. ICC values ST4

Table S5. Twin modeling values for full sample ST5

Table S6. Twin modeling values for cohabitation sample ST6

Table S7. Top 50 variants from EUR GWAS of the abundance of genus *Granulicatella*........ST7

Table S8. Top 50 variants from Meta-GWAS of the abundance of genus *Granulicatella*.......ST8

Table S9. Top 50 variants from Meta-GWAS of Unweighted Unifrac PCo3.........................ST9

Table S10. Top 50 variants from EUR GWAS of the abundance of genus *Granulicatella* with tobacco/marijuana/alcohol use included as covariate ST10

Table S11. Description of twin sample trait..ST11

Table S12. Description of twin sample traits limited to cohabitating sample ST12

Table S13. Description of European sample traits ST13

Table S14. Description of Admixture American sample traits ST14

Table S15. Comparison of GCTA and twin model heritability estimates.........................ST15

Table S16. Comparison twin heritability vs other studies.......................................ST16

Supplemental Figure 1. Abundant OTU table beta diversity comparisons: Beta diversity measurements for a. MZ(n=366 pairs), DZ(n=386 pairs), and unrelated individuals(n= 37832 pairs) and b. same sex DZ (n=263 pairs) and opposite sex DZ (n=123 pairs) using an OUT table that was filtered to contain only abundant OTUs (rarefied at 2500 reads and then filtered to OTUs present in at least 50% of the sample). Mann-Whitney U two-tailed test was applied between groups for Bray-Curtis, Weighted UniFrac, and Unweighted UniFrac Beta Diversity Measurements. P-values were determined with 10000 permutations of the group labels. (**) p-value<0.0005, (*) p-value<0.05

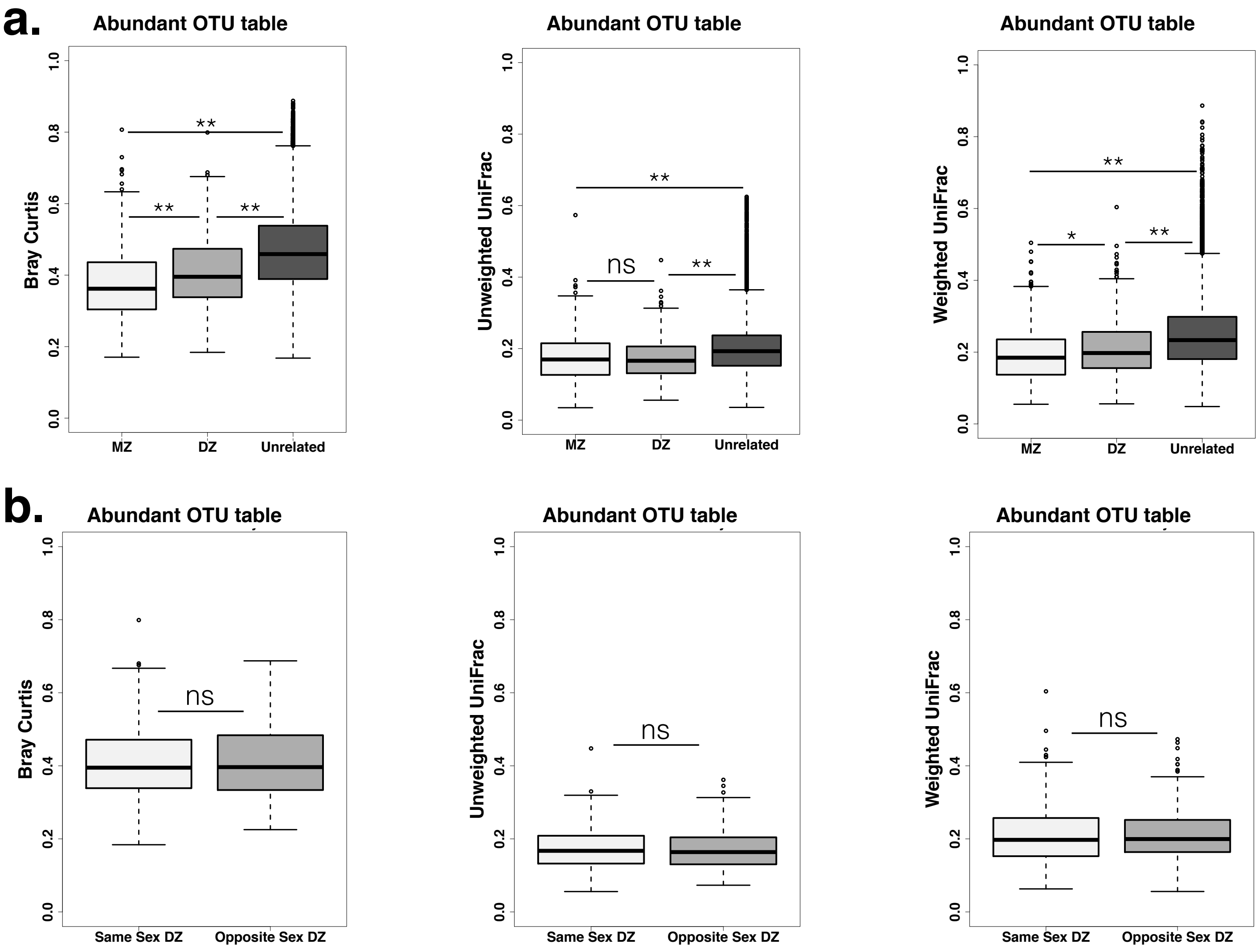

Supplemental Figure 2. Unrarefied and unfiltered OTU table beta diversity comparisons: Beta diversity measurements for a. MZ(n=366 pairs), DZ(n=386 pairs), and unrelated individuals(n= 37832 pairs) and b. same sex DZ (n=263 pairs) and opposite sex DZ (n=123 pairs) using an OUT table was not rarefied or filtered. Mann-Whitney U two-tailed test was applied between groups for Bray-c.Weighted UniFrac, and Unweighted UniFrac Beta Diversity Measurements. P-values were determined with 10000 permutations of the group labels. (**) p-value<0.0005, (*) p-value<0.05

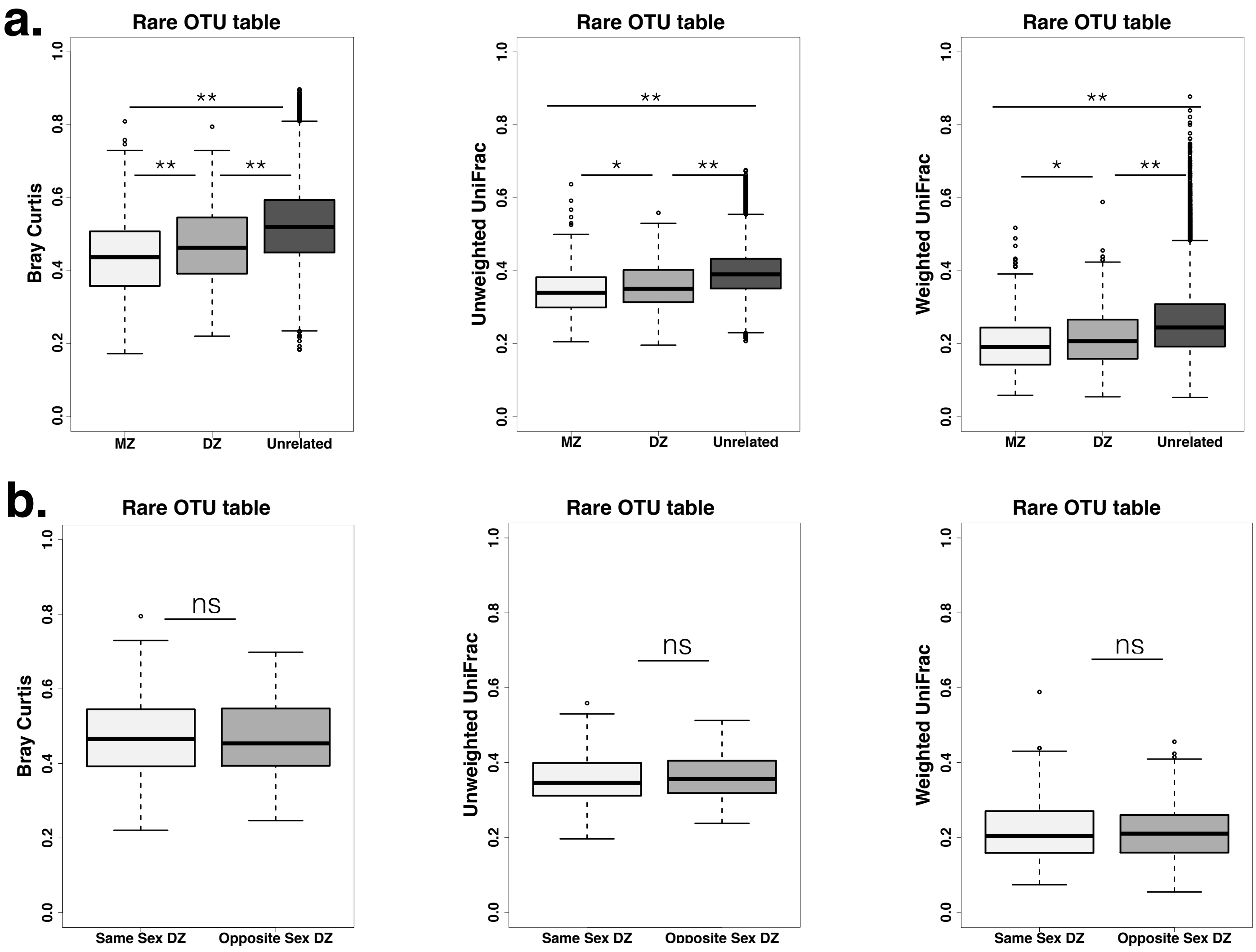

Supplemental Figure 3. Abundant OTU tables beta diversity comparisons limited to cohabitating twin pairs: Beta diversity measurements with only twin pairs either 18 and younger or those over the age of 18 and living together for a. MZ(n=294 pairs), DZ(n=294 pairs), and unrelated individuals(n= 37832 pairs) and b. same sex DZ (n=217 pairs) and opposite sex DZ (n=77 pairs) using an OUT table that was filtered to contain only abundant OTUs (rarefied at 2500 reads and then filtered to OTUs present in at least 50% of the sample). Mann-Whitney U two-tailed test was applied between groups for Bray-Curtis, Weighted UniFrac, and Unweighted UniFrac Beta Diversity Measurements. P-values were determined with 10000 permutations of the group labels. (**) p-value<0.0005, (*) p-value<0.05

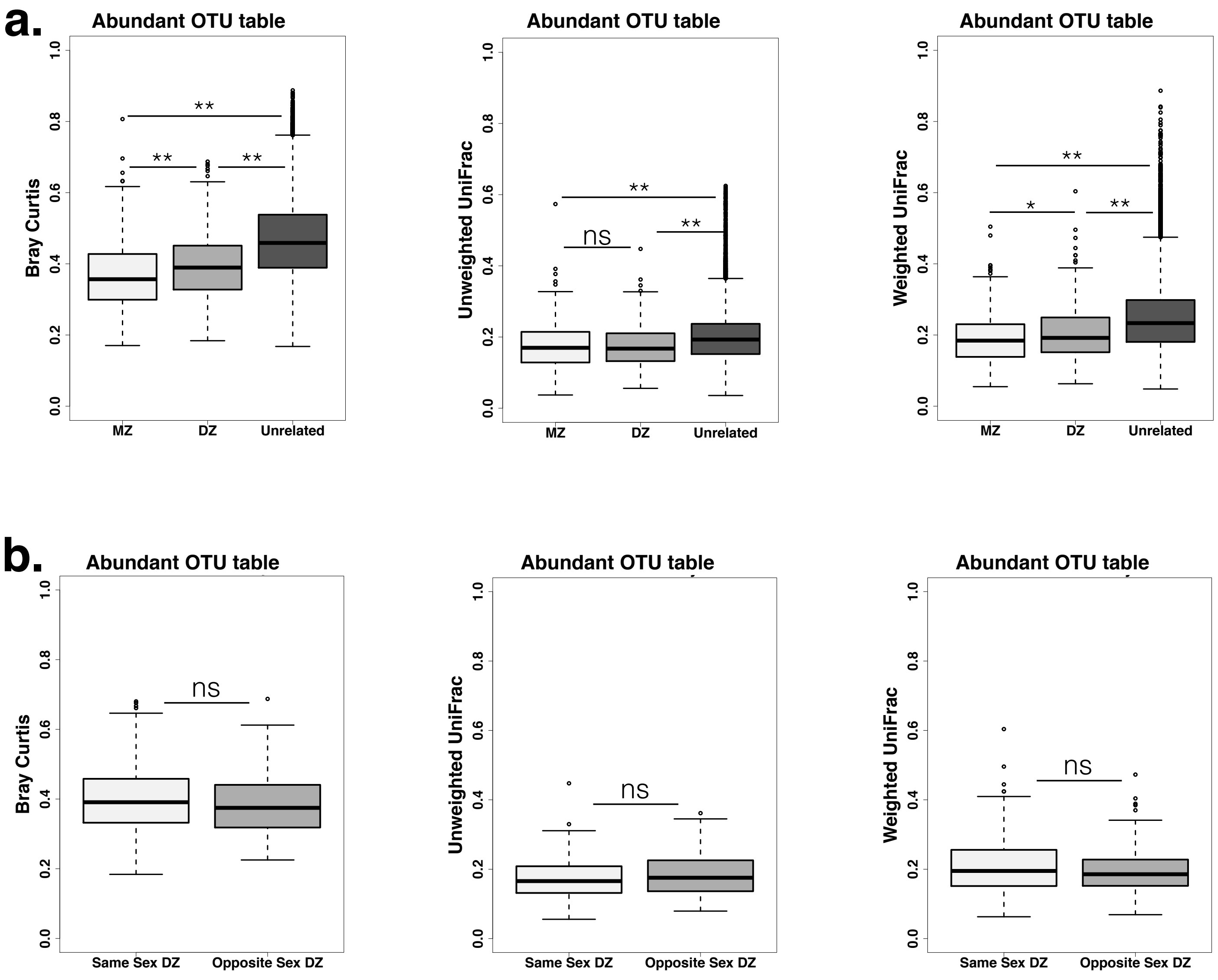

Supplemental Figure 4. Unrarefied and unfiltered OTU tables beta diversity comparisons limited to cohabitating twin pairs: Beta diversity measurements with only twin pairs either 18 and younger or those over the age of 18 and living together for a. MZ(n=294 pairs), DZ(n=294 pairs), and unrelated individuals(n= 37832 pairs) and b. same sex DZ (n=217 pairs) and opposite sex DZ (n=77 pairs) using an OUT table was not rarefied or filtered. Mann-Whitney U two-tailed test was applied between groups for Bray-Curtis, Weighted UniFrac, and Unweighted UniFrac Beta Diversity Measurements. P-values were determined with 10000 permutations of the group labels. (**) p-value<0.0005, (*) p-value<0.05

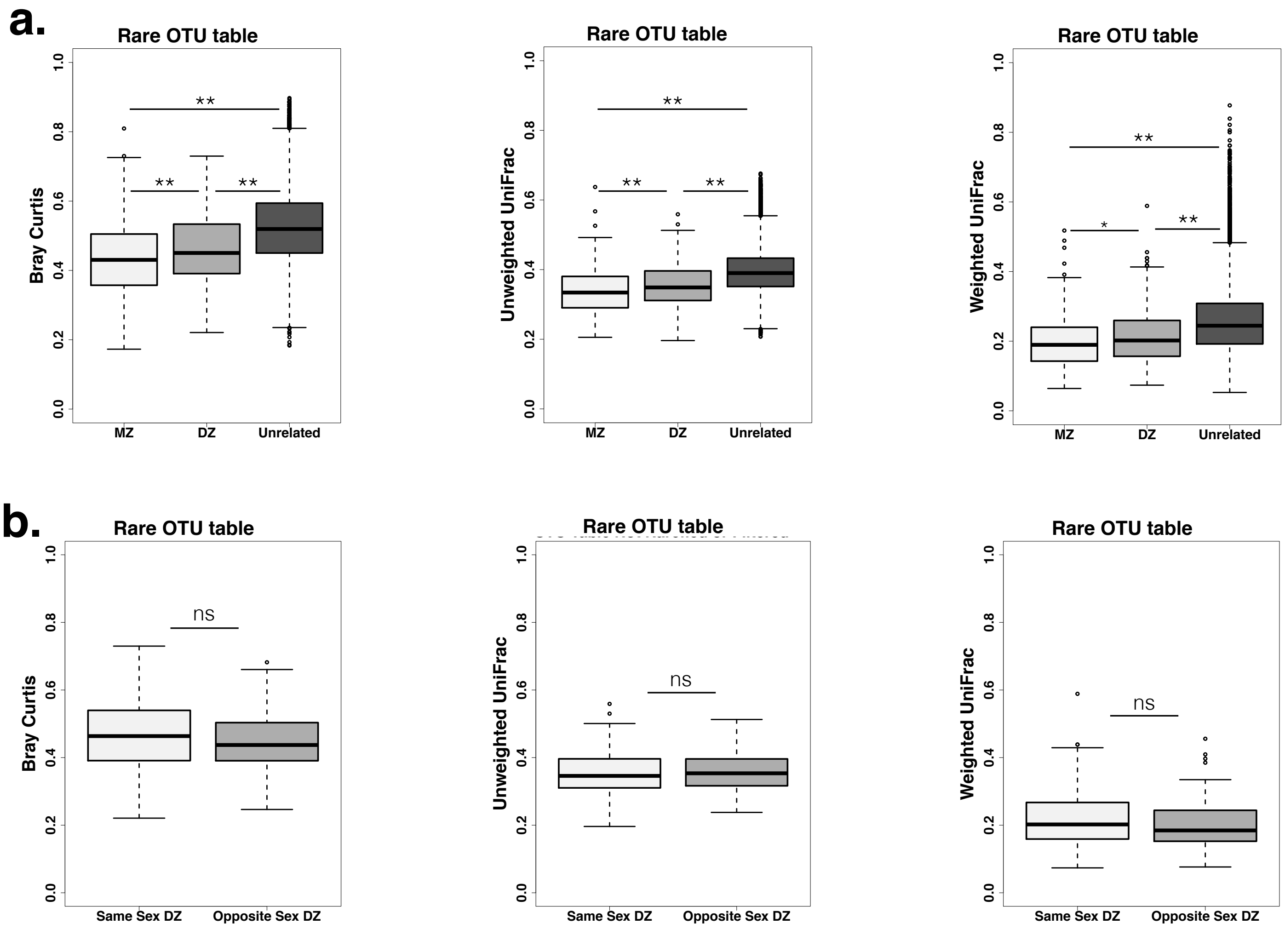

Supplemental Figure 5. Rarefaction comparison: Beta diversity measurements for MZ(n=366 pairs), DZ(n=386 pairs), and unrelated individuals(n= 37832 pairs) across four different rarefactions(2500 sequences per sample). Mann-Whitney U two-tailed test was applied between groups for Bray-Curtis, Weighted UniFrac, and Unweighted UniFrac Beta Diversity Measurements. P-values were determined with 10000 permutations of the group labels. (**) p-value<0.0005, (*) p-value<0.05

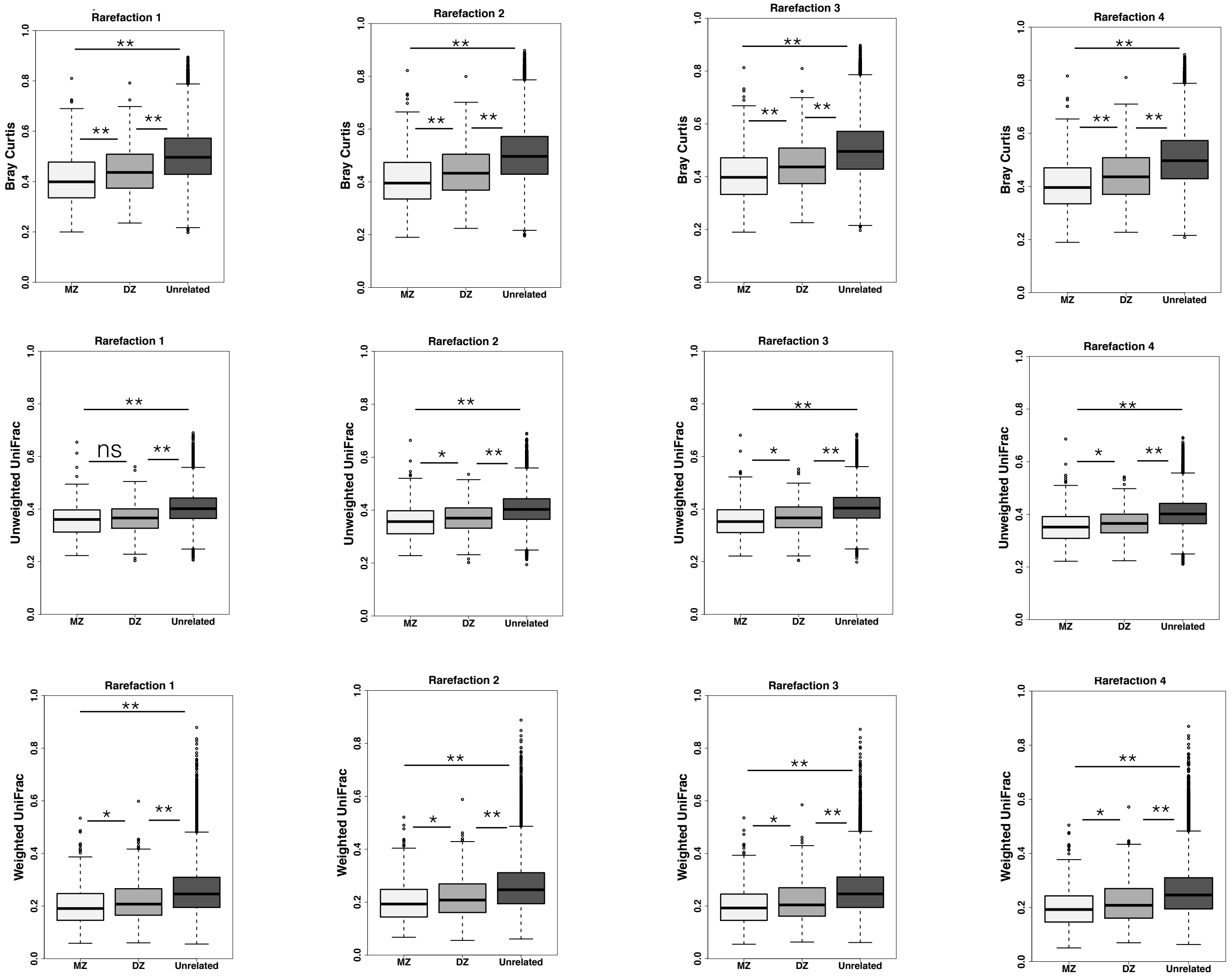

Supplemental Figure 6. Dizygotic opposite sex vs same sex pairs comparison: Beta diversity measurements for same sex DZ (n=263 pairs) and opposite sex DZ (n=123 pairs). Mann-Whitney U two-tailed test was applied between groups for Bray-Curtis, Unweighted and Weighted UniFrac Beta Diversity Measurements. P-values were determined with 10000 permutations of the group labels. (ns) not significant.

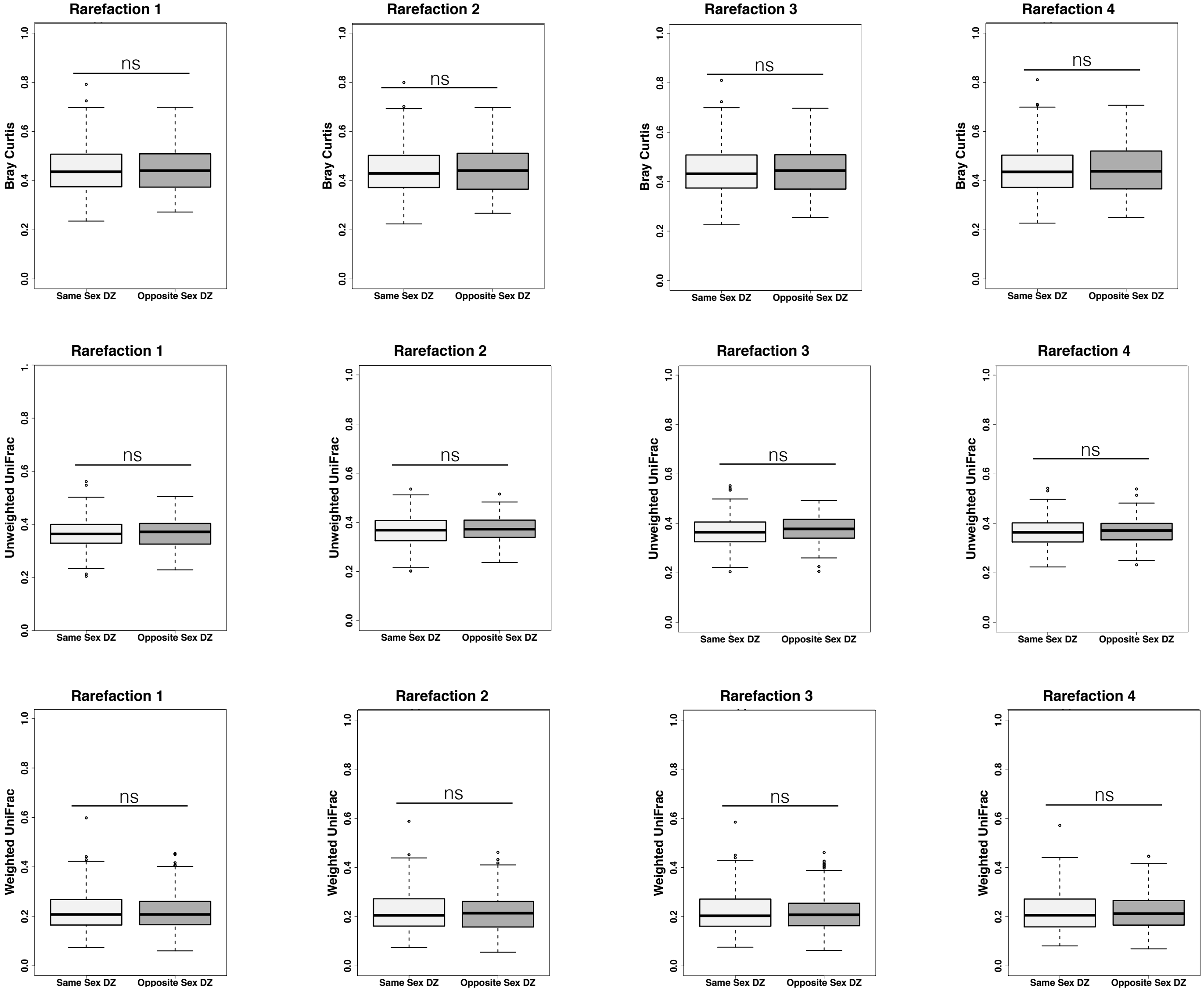

Supplemental Figure 7. Longitudinal samples: Beta diversity measurements for longitudinal pairs (56 pairs), and unrelated individuals who were not of the same age so as to match the longitudinal samples(15765 pairs). Mann-Whitney U two-tailed test was applied between groups. P-values were determined with 10000 permutations of the group labels (**) p-value<0.001, (*) p-value<0.01.

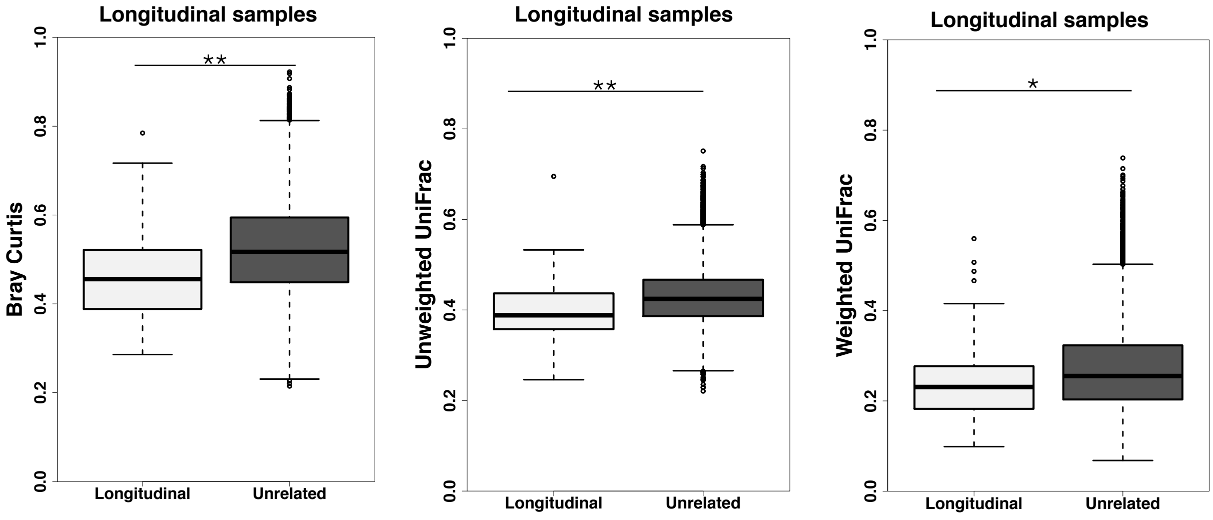

Supplemental Figure 8. Dizygotic opposite sex vs same sex pairs comparison with ICC: Intraclass correlation coefficient values for same sex DZ pairs (DZ, n=263 pairs) and opposite sex DZ twin pairs (OS, n=123 pairs) of taxa abundances. Taxa are grouped by phyla in. The ICC values between DZ and OS twin pairs were compared with the Wilcoxon Signed Rank Test with 10,000 permutations of the zygosity labels (Pvalue= 0.5591441).

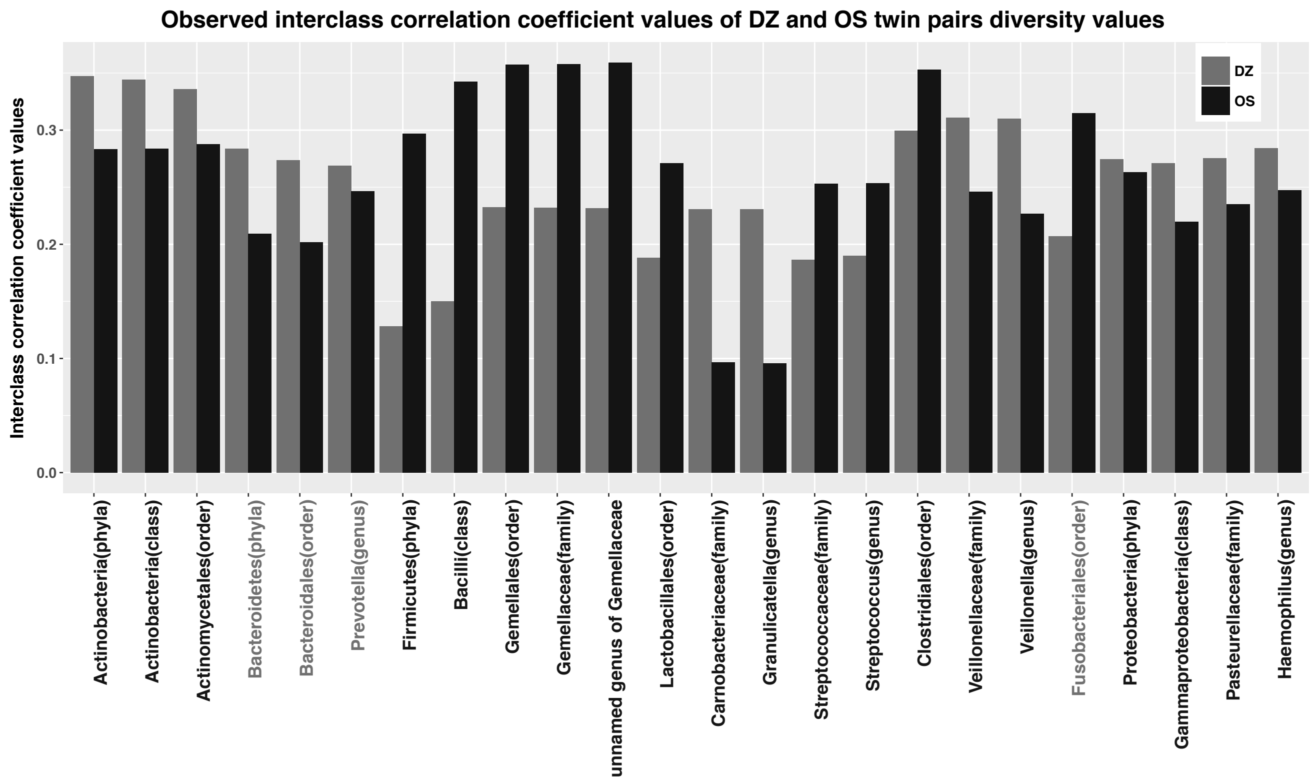

Supplemental Figure 9. Twin model power analysis: Power analyses for univariate twin modeling comparing the use of a continuous trait vs a binary trait of various prevalence's. Calculations completed in R. Black dashed verticle line is at 752 (full sample) and blue dashed vertical line is at 588 (cohabitation sample).

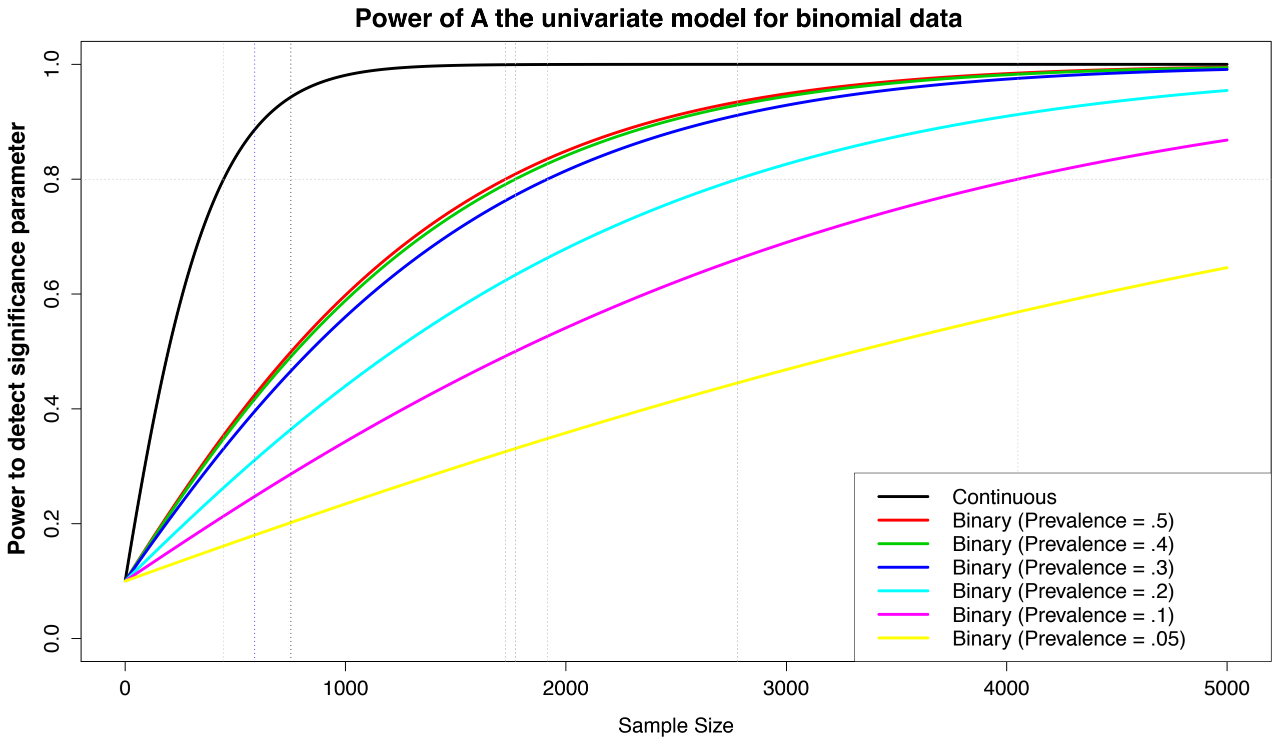

Supplemental Figure 10. Twin model average heritability estimates: Averaging heritability estimates (A) for traits within each phenotype category: alpha diversity, beta diversity PCos, OTUs, and taxanomic groups. a. Full sample (n=752 twin pairs) b. only twin pairs either 18 and younger or those over the age of 18 and living together, (n=588 twin pairs)

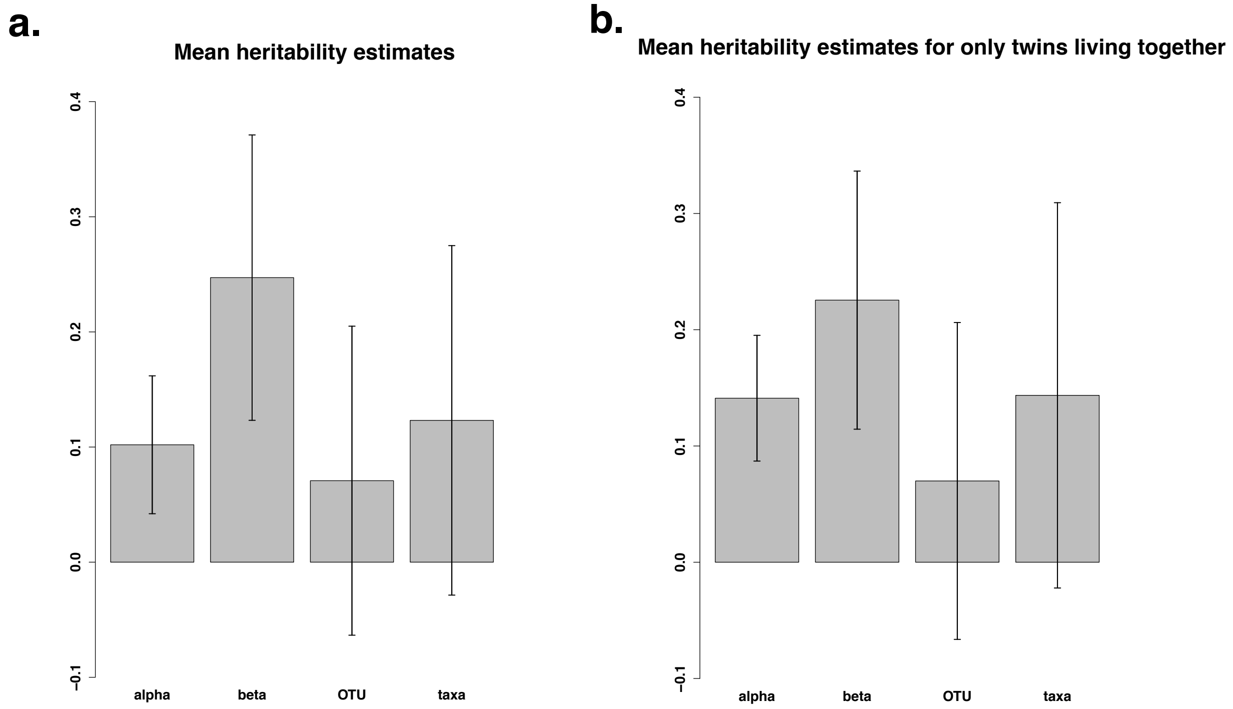

Supplemental Figure 11. Twin model pie charts: Pie chart of twin model estimates averaged across all phenotypes for a. Full sample (n=752 twin pairs) b. only twin pairs either 18 and younger or those over the age of 18 and living together, (n=588 twin pairs)

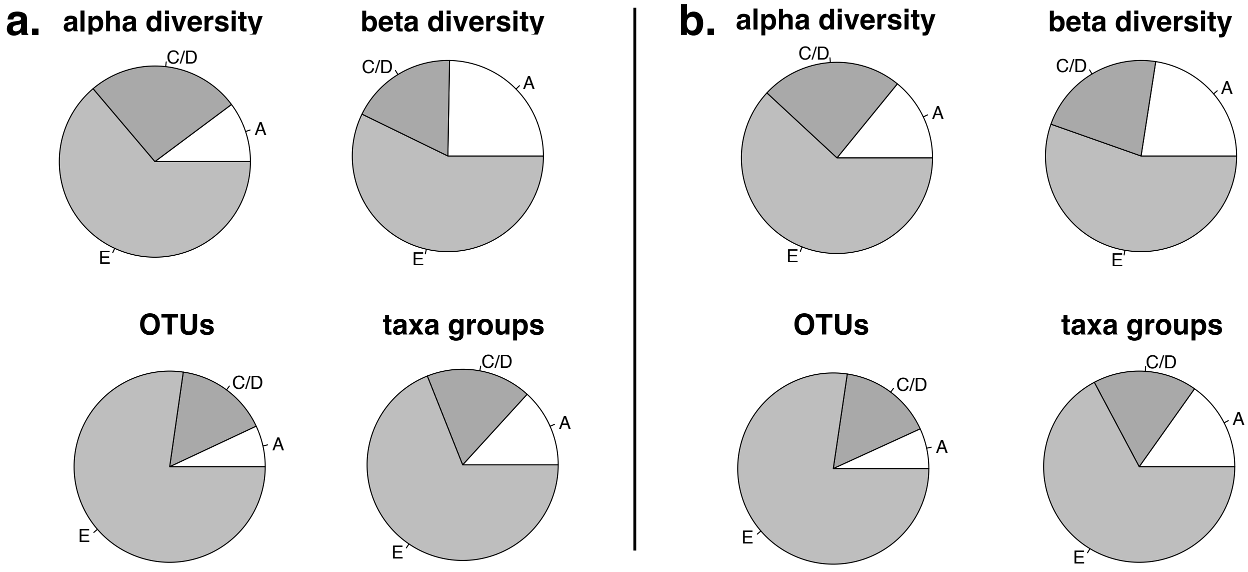

Supplemental Figure 12. Biplot analysis: Bray Curtis Principal Coordinate Biplot of all genre (n=752 pairs). The size of the genre sphere reflects its abundance, showing the genus Streptococcus is the most abundant. a. both subject and biplots displayed b. biplots only displayed

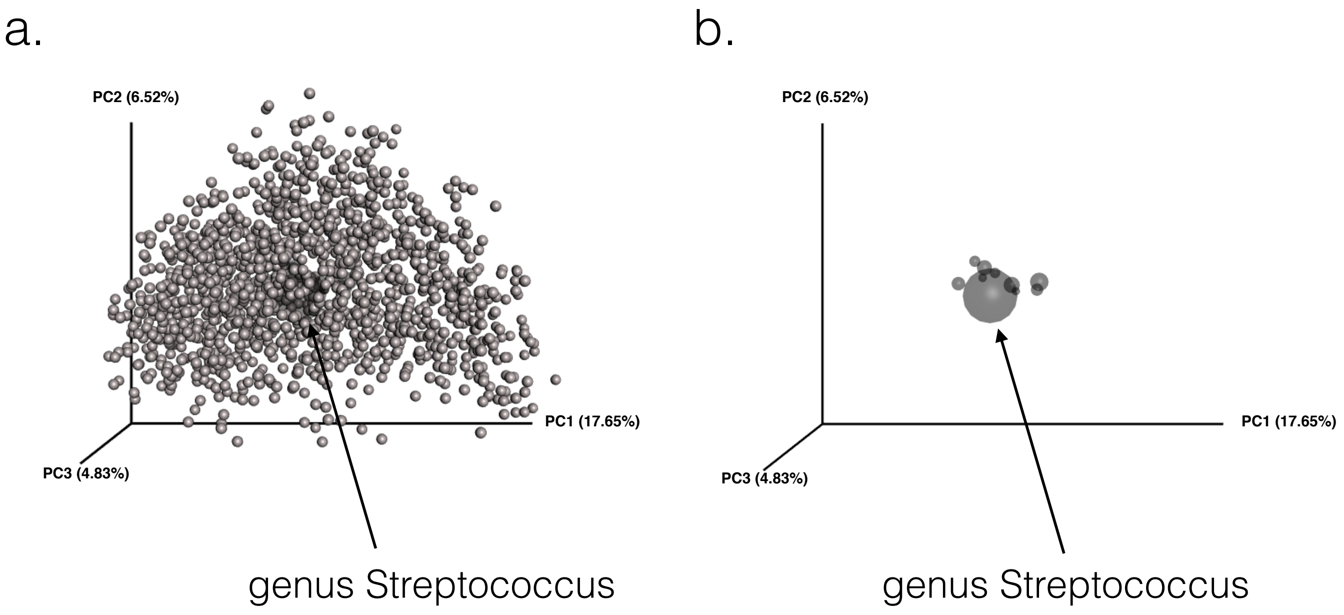

Supplemental Figure 13. Manhattan plots of European GWAS: Manhattan plot of the GWAS analysis in the European ancestry sample(n=823). The red line represents the threshold of genome wide significance(p-value<$5x10^{-8}$) The following phenotypes were transformed to z-scores and used in the GWAS (see Methods) a. Bray Curtis Principal Coordinate 2 b. Unweighted UniFrac Principal Coordinate 3 c. Abundance of genus *Granulicatella* d. Abundance of family *Veillonellacea* e. Weighed UniFrac Principal Coordinate 2 f. Unweighted UniFrac Principal Coordinate 2.

a. Manhattan plot in European sample Bray Curtis principal coordinate 2

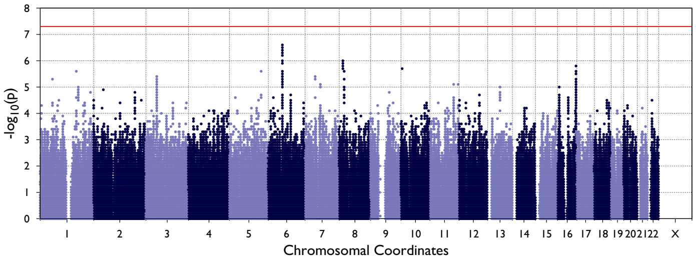

c. Manhattan plot in European sample genus *Granulicatella* abundance

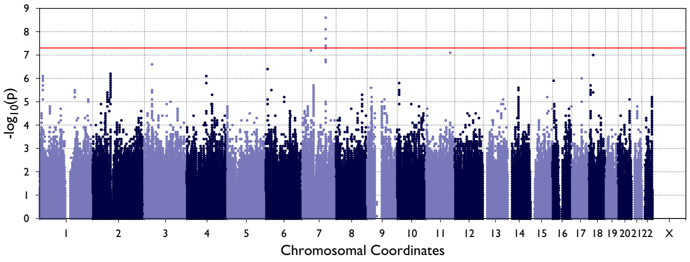

b. Manhattan plot in European sample Unweighted UniFrac principal coordinate 3

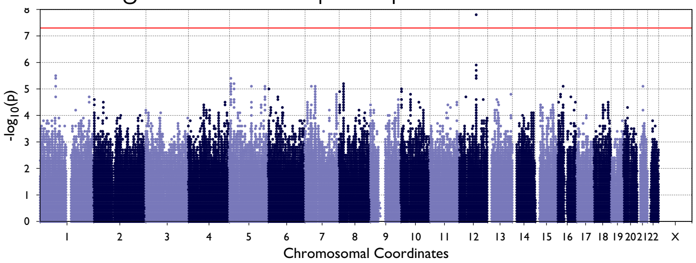

d. Manhattan Plot in European Sample family *Veillonellacea* Abundance

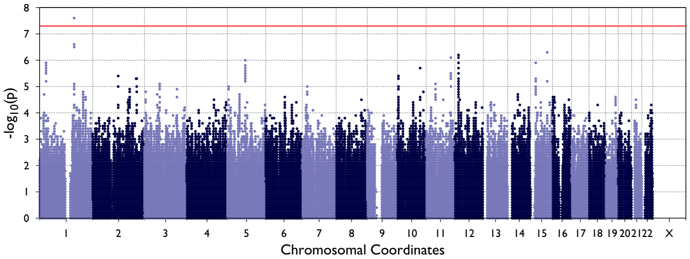

e. Manhattan plot in European sample Weighted UniFrac principal coordinate 2

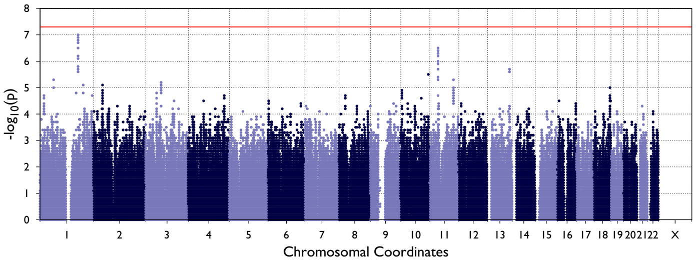

f. Manhattan plot in European sample Unweighted UniFrac principal coordinate 2

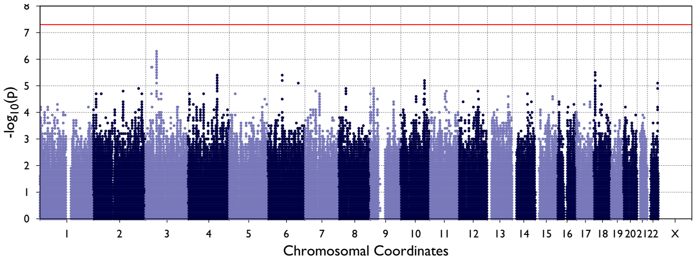

Supplemental Figure 14. QQ plots of the European GWAS: QQ Plot of the GWAS Analysis in the European ancestry sample(n=823). The following phenotypes were transformed to z-scores and used in the GWAS (see Methods) a. Bray Curtis Principal Coordinate 2 b. Unweighted UniFrac Principal Coordinate 3 c. Abundance of genus *Granulicatella* d. Abundance of family *Veillonellacea* e. Weighed UniFrac Principal Coordinate 2 f. Unweighted UniFrac Principal Coordinate 2.

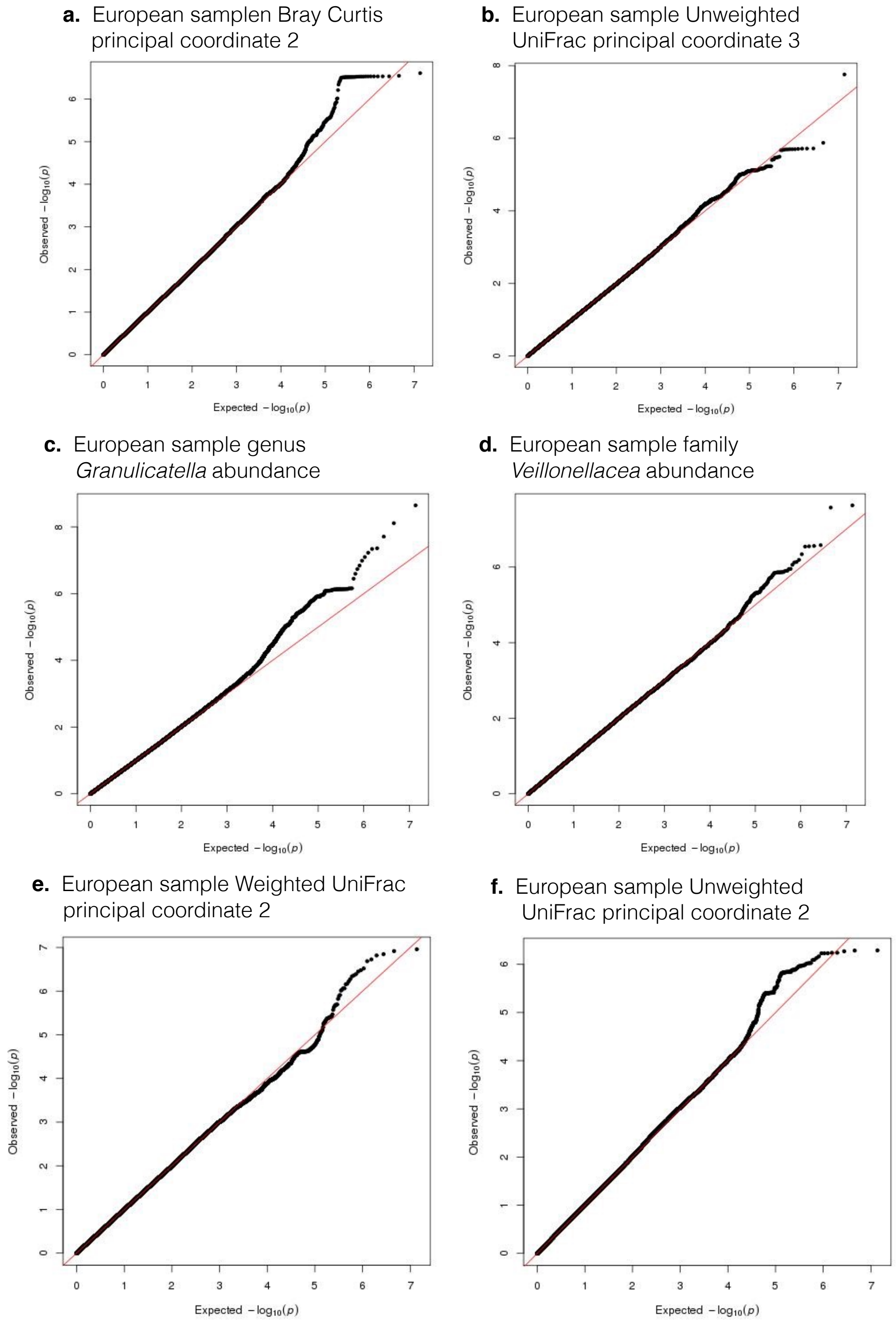

Supplemental Figure 15. Manhattan plots of European GWAS analysis with tobacco/marijuana/alcohol use as covariate: Manhattan plot of the GWAS analysis with tobacco/alcohol/marijuana use included as a covariate in the European ancestry sample(n=823). The red line represents the threshold of genome wide significance(p-value<5x10^{-8}) The following phenotypes were transformed to z-scores and used in the GWAS (see Methods) a. Bray Curtis Principal Coordinate 2 b. Unweighted UniFrac Principal Coordinate 3 c. Abundance of genus *Granulicatella* d. Abundance of family *Veillonellacea* e. Weighed UniFrac Principal Coordinate 2 f. Unweighted UniFrac Principal Coordinate 2.

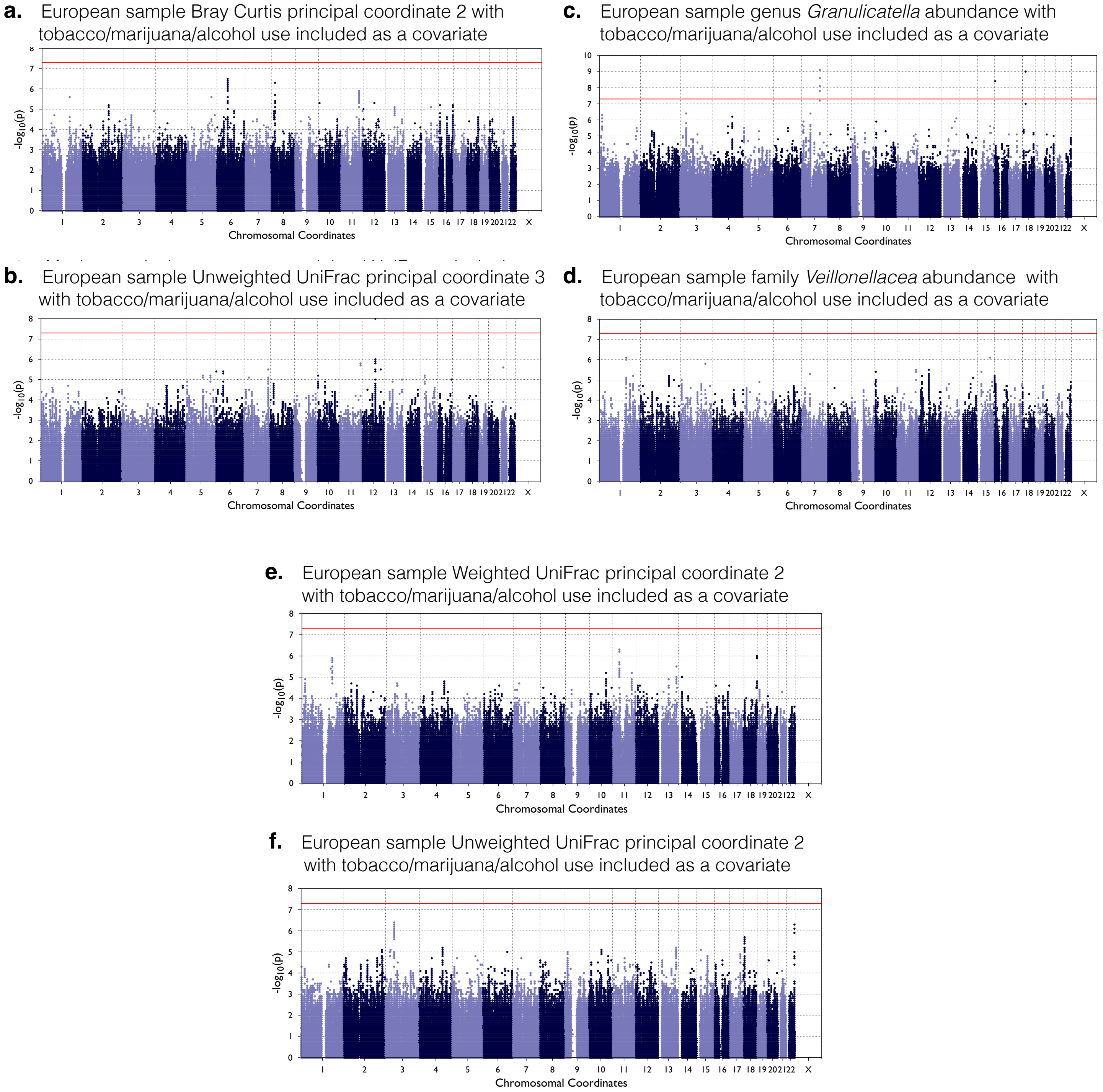

Supplemental Figure 16. QQ plots of European GWAS analysis with tobacco/marijuana/alcohol use as covariate: QQ Plot of the GWAS Analysis with tobacco/alcohol/marijuana use included as a covariate in the European ancestry sample(n=823). The following phenotypes were transformed to z-scores and used in the GWAS (see Methods) a. Bray Curtis Principal Coordinate 2 b. Unweighted UniFrac Principal Coordinate 3 c. Abundance of genus *Granulicatella* d. Abundance of family *Veillonellacea* e. Weighed UniFrac Principal Coordinate 2 f. Unweighted UniFrac Principal Coordinate 2.

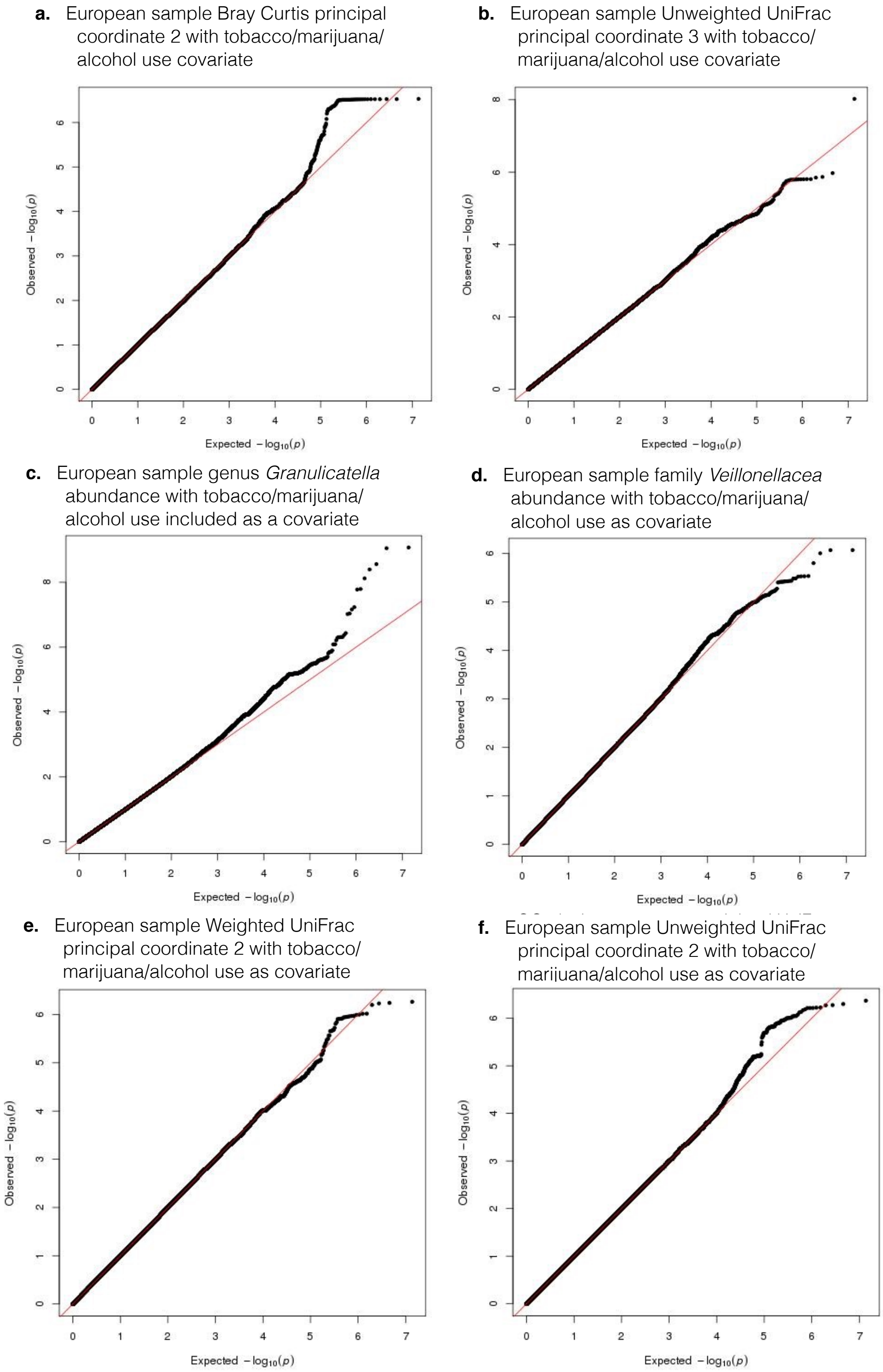

Supplemental Figure 17. Ancestry principal components: Ancestry Principal Components 1-10 created with LD pruned SNPs (see methods). a Admixture ancestry sample(n=344). b. European ancestry sample (n=823)

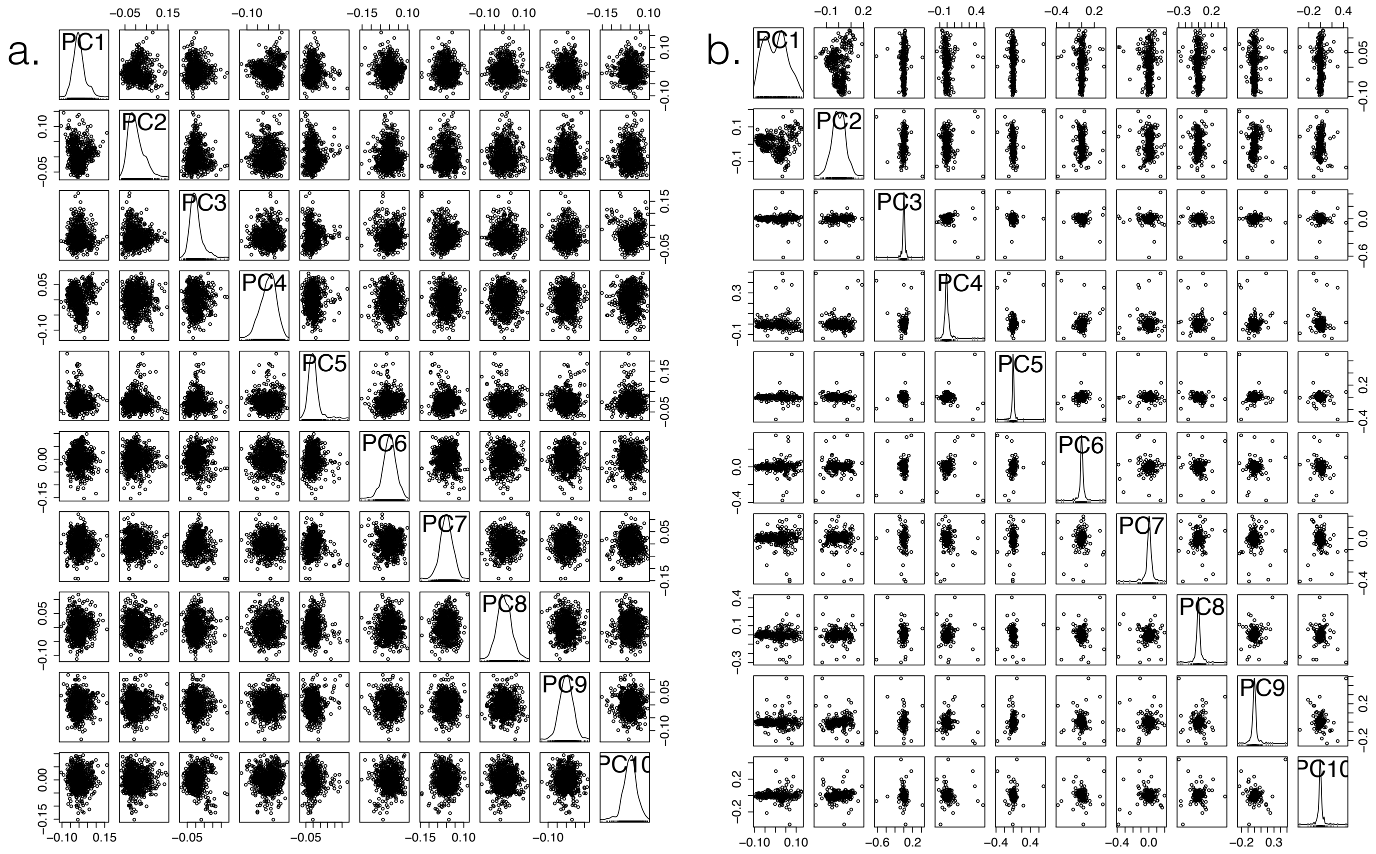

Supplemental Figure 18. QQ plots of Comparison SNPs: QQ Plot of the GWAS analyses with those SNPs that overlapped with previous microbiome studies colored in black, and those that did not overlap in red. a. Abundance of genus *Granulicatella* GWAS in Europeans. b. Abundance of genus *Granulicatella* GWAS in Europeans limited to only those SNPs that overlapped with previous studies. c. Unweighted UniFrac Principal Coordinate 3 Meta-Analysis. d. Unweighted UniFrac Principal Coordinate 3 Meta-Analysis limited to only those SNPs that overlapped with previous studies.

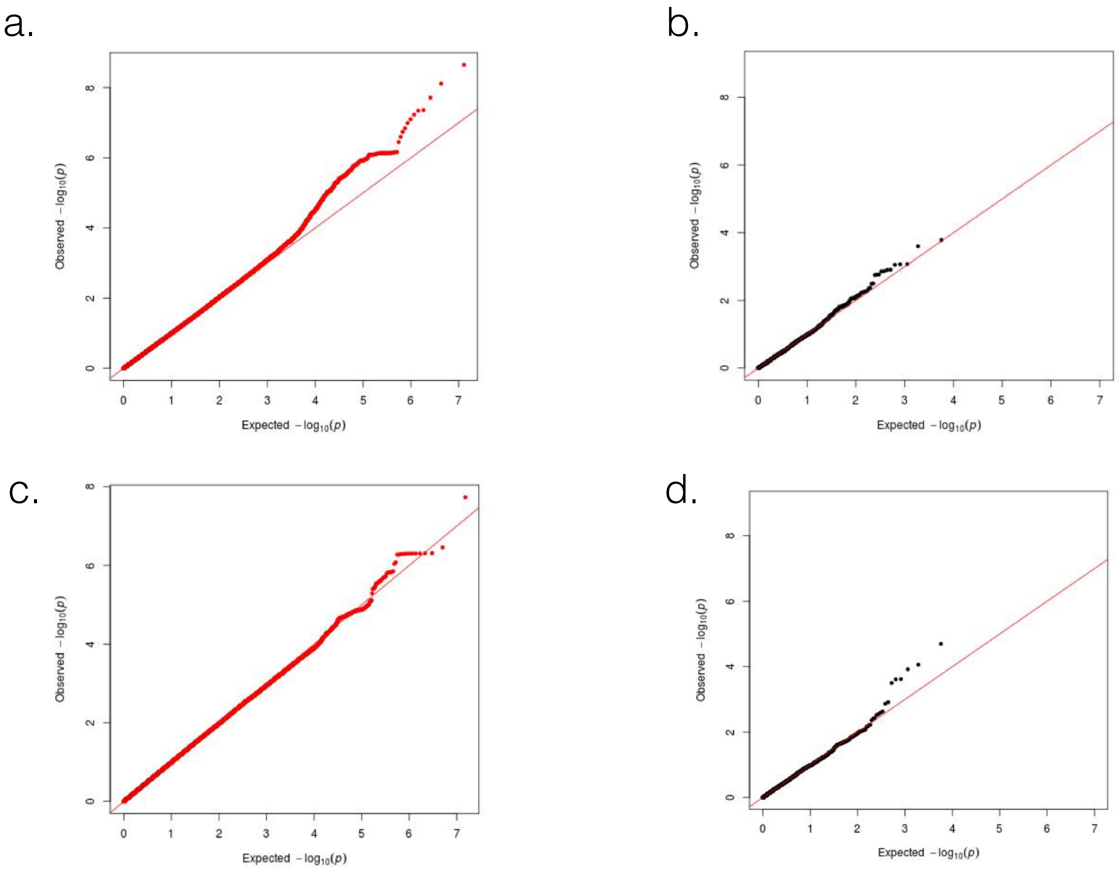

Supplemental Methods

ACE/ADE Twin Modeling

Traits were modeled either as continuous or as categorical via the threshold model depending on their specified class (Supplemental Table 11&12). The classification of each trait was completed using the entire twin sample and was used for both the full and cohabitation sample modeling. Selection of which model to use (ACE vs ADE) was determined within each population separately. For the continuous trait models we forced means to be equal across zygosity for all traits except the 6 which violated the assumption. For those that violated the assumption a separate mean was used for each zygosity class, which should only change the fit of the model but the estimates should remain consistent. Almost all continuous traits did not violate any assumptions, but for those that did we would expect only the fit of the model to change not the estimates (Supplemental Table 5-6). We also did a crude evaluation of the categorical traits but further analyses will be need. Due to skewness and variability in the categorical distributions caution should be taken when interpreting these effects. However we are confident that these analyses give insight into the effect of the traits. Only traits in which estimates from the twin model could be obtained are displayed in Supplementary Table 5&6, 11&12 (full sample= 946 traits, cohabitation sample=907 traits).

Twin Heritability Estimate Comparisons

The top 44 most heritable traits defined by a Benjamin-Hochberg corrected P value of less than 1 were compared to heritability estimates provided in the following tables: Corby et al 2007 (Table 3, heritability estimates from all subjects (Corby et al., 2007)), Goodrich et al 2016 (Table S1, Table S2,(Goodrich et al., 2016)), Davenport et al 2015 (Table S2, (Davenport et al., 2015)), and Turpin et al 2016 (Table S2, (Turpin et al., 2016)). A summary of the 44 traits compared is in Supplementary Table 16.

Genome Complex Trait Analysis Dissatenuated Correlation

Traits that were categorized as continuous in both the twin sample and European population were compared (n=40 traits, see Methods, Supplementary Tables 11,13,15). The dissatenuated correlation was calculated using the formula r(x,y)/sqrt(r(x,x),r(y,y)) where x= GCTA_h2 and y = ACE_A, each of which are a vector of either GCTA or standardized A estimates(Spearman, 1904, 1910). The denominator in the equation above is derived to be [sqrt(var(GCTA_h2) - SE(GCTA_h2)^2)*sqrt(var(ACE_h2) - SE(ACE_h2)^2)] / [sqrt(var(GCTA_h2)*var(ACE_h2))] where SE(GCTA_h2) is 316/N for all estimates and SE(ACE_h2) is the standard error for A estimates in ACE models(Visscher et al 2014). The mean GCTA heritability estimate and standard errors was calculated from all traits categorized as continuous in the European population (n=55 traits, see Methods, Supplementary Table 13). P values estimated in GCTA were corrected for multiple the multiple testing of 55 traits using the Benjamin-Hochberg method (p.adjust in the stats package in R)(Benjamini and Hochberg, 1995).

Substance Use Covariates

Substance use information for the past six months was available for 92% of the subjects. Tobacco use was scored 0=no tobacco use in the past 180 days, 1=tobacco used 1-179 days past 180 days, 2=tobacco used every day in the past 180 days. Marijuana use was scored 0=no marijuana use in the past 180 days, 1=marijuana used 1-49 days past 180 days, 2=marijuana used 50-180 days past 180 days. Alcohol use was scored 0=no alcohol use in the past 180 days, 1=alcohol used 1-99 days past 180 days, 2=alcohol used 100-180 days past 180 days.

Genome Wide Association Study Comparisons

SNPs from the abundance of genus *Granulicatella* GWAS and Unweighted UniFrac PC3 Meta-Analysis were selected (for which rs numbers could be obtained). Within the abundance of genus Granulicatella and Unweighted UniFrac PC3 SNPs if there were duplicates present one was randomly chosen, resulting in a total 6405171 SNPs and 7573117 respectively. These were compared to 3983 unique SNPs collated from the following studies. Bleckhman et al 2015 (Table S5,(Blekhman et al., 2015)), Goodrich et al 2016 (Table 2, Table S5,(Goodrich et al., 2016)), Davenport et al 2015 (Table S7,(Davenport et al., 2015)), Bonder et al 2016 (Table in Figure 2, Table S3, Table S7, Table S9, Table S10, Table S11, Table S12,(Bonder et al., 2016)), Turpin et al 2016 (Table S3, Table S4, Table S5, Table S6,(Turpin et al., 2016)), and Wang et al (Table 1, Table 2, Table S2, Table S5, Table S6, Table S7,(Wang et al., 2016)). There were 2826 SNPs that overlapped with the abundance of g genus *Granulicatella* GWAS and 2867 with the Unweighted UniFrac PC3. These SNPs were then plotted on a QQ-plot using the P value from the abundance of g genus *Granulicatella* GWAS or Unweighted UniFrac PC3 Meta-Analysis respectively using the “qqman” package in R (R Core Team, 2015; Turner, 2014).

Knowledge-based mining system for Genome-Wide Genetic studies

The program Knowledge-based mining system for Genome-Wide Genetic studies (KGG) was used to perform the gene-based analyses test(Li et al., 2010).The European reference panel provided on the KGG website was used as the reference with default settings. While the meta-GWAS results did include the Admixture American population, because the majority of the sample was European the European reference panel was still used. Corrected p-value were calculated the Benjamin-Hochberg method (default settings)(Benjamini and Hochberg, 1995).

References

Benjamini, Y., and Hochberg, Y. (1995). Controlling the False Discovery Rate: A Practical and Powerful Approach to Multiple Testing. J. R. Stat. Soc. Ser. B Methodol. *57*, 289–300.

Blekhman, R., Goodrich, J.K., Huang, K., Sun, Q., Bukowski, R., Bell, J.T., Spector, T.D., Keinan, A., Ley, R.E., Gevers, D., et al. (2015). Host genetic variation impacts microbiome composition across human body sites. Genome Biol. *16*, 191.

Bonder, M.J., Kurilshikov, A., Tigchelaar, E.F., Mujagic, Z., Imhann, F., Vila, A.V., Deelen, P., Vatanen, T., Schirmer, M., Smeekens, S.P., et al. (2016). The effect of host genetics on the gut microbiome. Nat. Genet. *48*, 1407–1412.

Corby, P.M., Bretz, W.A., Hart, T.C., Schork, N.J., Wessel, J., Lyons-Weiler, J., and Paster, B.J. (2007). Heritability of oral microbial species in caries-active and caries-free twins. Twin Res. Hum. Genet. Off. J. Int. Soc. Twin Stud. *10*, 821–828.

Davenport, E.R., Cusanovich, D.A., Michelini, K., Barreiro, L.B., Ober, C., and Gilad, Y. (2015). Genome-Wide Association Studies of the Human Gut Microbiota. PloS One *10*, e0140301.

Goodrich, J.K., Davenport, E.R., Beaumont, M., Jackson, M.A., Knight, R., Ober, C., Spector, T.D., Bell, J.T., Clark, A.G., and Ley, R.E. (2016). Genetic Determinants of the Gut Microbiome in UK Twins. Cell Host Microbe *19*, 731–743.

Li, M.-X., Sham, P.C., Cherny, S.S., and Song, Y.-Q. (2010). A Knowledge-Based Weighting Framework to Boost the Power of Genome-Wide Association Studies. PLOS ONE *5*, e14480.

R Core Team (2015). R: A language and environment for statistical computing (Vienna, Austria: R Foundation for Statistical Computing).

Spearman, C. (1904). The Proof and Measurement of Association between Two Things. Am. J. Psychol. *15*, 72–101.

Spearman, C. (1910). Correlation Calculated from Faulty Data. Br. J. Psychol. 1904-1920 *3*, 271–295.

Turner, S.D. (2014). qqman: an R package for visualizing GWAS results using Q-Q and manhattan plots. bioRxiv 5165.

Turpin, W., Espin-Garcia, O., Xu, W., Silverberg, M.S., Kevans, D., Smith, M.I., Guttman, D.S., Griffiths, A., Panaccione, R., Otley, A., et al. (2016). Association of host genome with intestinal microbial composition in a large healthy cohort. Nat. Genet. *48*, 1413–1417.

Wang, J., Thingholm, L.B., Skiecevičienė, J., Rausch, P., Kummen, M., Hov, J.R., Degenhardt, F., Heinsen, F.-A., Rühlemann, M.C., Szymczak, S., et al. (2016). Genome-wide association analysis identifies variation in vitamin D receptor and other host factors influencing the gut microbiota. Nat. Genet. *48*, 1396–1406.

Supplementary Tables

Supplementary Table 1. Cohen's D effect size for full sample: Cohen's d effect size and category calculated with the full sample (n=752 twin pairs) for Bray Curtis, Weighted UniFrac, and Unweighted UniFrac (see Methods).

Supplementary Table 2. Cohen's D effect size limited to cohabitating twin pairs: Cohen's d effect size and category calculated with only twin pairs either 18 and younger or those over the age of 18 and living together (n=588 twin pairs) for Bray Curtis, Weighted UniFrac, and Unweighted UniFrac (see Methods).

Supplementary Table 3. Cohen's D effect size full sample vs cohabitating twin pair: Cohen's d effect size and category calculated for the full sample (n=752 twin pairs) vs the cohabitation sample (only twin pairs either 18 and younger or those over the age of 18 and living together, n-588 twin pairs) for Bray Curtis, Weighted UniFrac, and Unweighted UniFrac (see Methods).

Supplementary Table 4. ICC values: Intraclass Correlation Coefficient for all twin pairs MZ(n=386 pairs), DZ (all DZ, n=366 pairs), DZ same sex only (n=263 pairs), DZ opposite sex only (n=123 pairs) for all 56 continuous traits. The columns are as follows: *TwinTrait*= trait coded name (see Supplementary Table 11), *ICC_MZ*=intraclass correlation coefficient values for MZ pairs, *ICC_DZ*=intraclass correlation coefficient values for DZ pairs(all DZ twin pairs), ICC*_DZ _same_sex_only*=intraclass correlation coefficient values for same sex DZ pairs), *ICC_DZ_opposite_sex_only*=intraclass correlation coefficient values for opposite sex DZ pairs,*Taxa*=trait is a taxon class and was included in Figure 2.

Supplementary Table 5. Twin modeling values for full sample: Twin Modeling Values calculated using the full sample (n=752 twin pairs)(see Methods). The columns are as follows: *TwinTrait*= trait coded name (see Supplementary Table 11), *TraitCategory*=continuous vs categorical, *TwinMode*l= ACE vs ADE, *StandardizedA_Heritability*= standardized A value ("heritability"), *StandardizedALowerCI*= lower 95% confidence interval (CI) bound for Standardized A, *StandardizedAUpperCI*= upper 95% CI for standardized A , *StandardizedAPValue_estimated_from_twinmodel*= pvalue for Standardized A determined from the twin model, *StandardizedE*= standardized E value, *StandardizedELowerCI* =lower 95% CI for standardized E , *StandardizedEUpperCI*= upper 95% CI for standardized E , *StandardizedCD*= standardized C or D (depending on the model specified in TwinModel), *StandardizedCDLowerCI* =lower 95% CI for standardized C or D, *StandardizedCDUpperCI*= lower 95% CI for standardized C or D, *AssumptionTestPvalue_ContinuousTraits_EqualMeansAcrossTwinOrder*=P value from model testing equal means across twin order (P value<0.05 suggests assumption violation)*,* *AssumptionTestPvalue_ContinuousTraits_EqualMeansAndVarianceAcrossTwinOrder*=P value from model testing equal means and variance across twin order (P value <0.05 suggests assumption

violation),*AssumptionTestPvalue_ContinuousTraits_EqualMeansAndVarianceAcrossTwinOrderAndZygosity*=P value from model testing equal means and variance across twin order and zygosity (P value <0.05 suggests assumption violation), *AssumptionTestPvalue_CategoricalTraits_EqualThresholdsAcrossTwinOrder*=Pvalue from model testing equal threshold across twin order (P value <0.05 suggests assumption violation),*AssumptionTestPvalue_CategoricalTraits_EqualThresholdsAcrossTwinOrderAndZygosity*=P value from model testing equal threshold across twin order and zygosity (P value <0.05 suggests assumption violation), *CorrectedStandardizedAPValue*= corrected pvalue for standardized A (correcting for 946 tests using the p.adjust package in R (Benjamin&Hochberg method))

Supplementary Table 6. Twin modeling values for cohabitation sample: Twin Modeling Values calculated using only twin pairs either 18 and younger or those over the age of 18 and living together (n=588 twin pairs see Methods). The columns are as follows: *TwinTrait*= trait coded name (see Supplementary Table 12), *TraitCategory*=continuous vs categorical, *TwinModel*= ACE vs ADE, *StandardizedA_Heritability*= standardized A value ("heritability"), *StandardizedALowerCI*= lower 95% confindence interval (CI) bound for Standardized A, *StandardizedAUpperCI*= upper 95% CI for standardized A , *StandardizedAPvalue_estimated_from_twinmodel*= P value for Standardized A deteremined from the twin model, *StandardizedE*= standardized E value, *StandardizedELowerCI* =lower 95% CI for standardized E , *StandardizedEUpperCI*= upper 95% CI for standardized E , *StandardizedCD*= standardized C or D (depending on the model specified in TwinModel), *StandardizedCDLowerCI* =lower 95% CI for standardized C or D, *StandardizedCDUpperCI*= lower 95% CI for standardized C or D, *AssumptionTestPvalue_ContinuousTraits_EqualMeansAcrossTwinOrder*=P value from model testing equal means across twin order (P value<0.05 suggests assumption violation), *AssumptionTestPvalue_ContinuousTraits_EqualMeansAndVarianceAcrossTwinOrder*= P value from model testing equal means and variance across twin order (P value<0.05 suggests assumption violation),*AssumptionTestPvalue_ContinuousTraits_EqualMeansAndVarianceAcrossTwinOrderAndZygosity*=P value from model testing equal means and variance across twin order and zygosity (P value<0.05 suggests assumption violation), *AssumptionTestPvalue_CategoricalTraits_EqualThresholdsAcrossTwinOrder*=P value from model testing equal threshold across twin order (P value<0.05 suggests assumption violation),*AssumptionTestPvalue_CategoricalTraits_EqualThresholdsAcrossTwinOrderAndZygosity*=P value from model testing equal threshold across twin order and zygosity(P value <0.05 suggests assumption violation), *CorrectedStandardizedAPValue*= corrected P value for standardized A (correcting for 907 tests using the p.adjust package in R (Benjamin&Hochberg method, p.adjust package in R))

Supplementary Table 7. Top 50 variants from EUR GWAS of the abundance of genus Granulicatella: Top 50 variants from the European population GWAS performed with EPACTS for the abundance of the genus Granulicatella. The columns are as follows: *CHROM*= chromosome location, *BEG*= base pair variant starts, *END*= base pair location variant ends,

MARKER_ID= description of variant (variant name,reference allele according to reference/ alternative allele according to reference, location description of variant),*AC*= allele count(dosage), *MAF*= minor allele frequency calculated for sample(uses sample counts to determine minor allele), *HWE_P*= Hardy-Weinberg equilibrium p value (determined from allele calls), *BETA*= beta from regression analyses (tests the reference alternative allele),SEBETA=standard error of beta, *R2*= r-squared value from regression analyses, *PVALUE*= P value from regression analyses

Supplementary Table 8. Top 50 variants from Meta-GWAS of the abundance of genus *Granulicatella*: Top 50 variants from the GWAS Meta Analyses performed with METAL for the abundance of the genus *Granulicatella*. The columns are as follows: *MarkerName*= variant name, *Allele1*= reference allele defined by reference, *Allele2*= alternative allele defined by reference, *Weight*= weight given to variant, *Zscore*= combined z-statistic for the variant , *PValue*= P value from METAL analyses corrected for genomic inflation, *Direction*=direction of variants effects within each study, *HWE_P_EUR*= Hardy-Weinberg equilibrium P value (determined from allele calls within European population), *HWE_P_ADM*= Hardy-Weinberg equilibrium P value (determined from allele calls within Admixture American population)

Supplementary Table 9. Top 50 variants from Meta-GWAS of Unweighted unifrac PCo3: Top 50 variants from the GWAS Meta Analyses performed with METAL for Unweighted UniFrac Principal Coordinate 3. The columns are as follows: *MarkerName*= variant name, *Allele1*= reference allele defined by reference, *Allele2*= alternative allele defined by reference, *Weight*= weight given to variant, *Zscore*= combined z-statistic for the variant , *PValue*= P value from METAL analyses corrected for genomic inflation, *Direction*=direction of variants effects within each study, *HWE_P_EUR*= Hardy-Weinberg equilibrium p value (determined from allele calls within European population), *HWE_P_ADM*= Hardy-Weinberg equilibrium P value (determined from allele calls within Admixture American population)

Supplementary Table 10. Top 50 variants from EUR GWAS of the abundance of genus *Granulicatella* with tobacco/marijuana/alcohol use included as covariate: Top 50 variants from the European population GWAS performed with EPACTS for the abundance of the genus *Granulicatella* with tobacco/marijuana/alcohol use included as a covariate. The columns are as follows: *CHROM*= chromosome location, *BEG*= base pair variant starts, *END*= base pair location variant ends, *MARKER_ID*= description of variant (variant name, reference allele according to reference/alternative allele according to reference, location description of variant),*AC*= allele count(dosage), *MAF*= minor allele frequency calculated for sample(uses sample counts to determine minor allele), *HWE_P*= Hardy-Weinberg equilibrium P value (determined from allele calls), *BETA*= beta from regression analyses (tests the reference alternative allele),SEBETA=standard error of beta, *R2*= r-squared value from regression analyses, *PVALUE*= P value from regression analyses

Supplementary Table 11. Description of twin sample trait: Description of twin sample traits as categorized within the full sample (n=752 twin pairs). The columns are as follows:

TwinTrait= trait number (see Supplementary Table 11), *TraitDescription*=full description of trait, *TaxonomyOfOTU*=taxonomy assignment if trait is OTU, *TraitType*=trait type of either alpha diversity, principal coordinate from beta diversity, OTU, taxonomic group, or collapsed OTU and taxonomic group, *TraitCategory*= trait category of either continuous or categorical.

Supplementary Table 12. Description of twin sample traits limited to cohabitating sample Description of twin sample traits as categorized within the cohabitation sample, limited to twins cohabitating together (n=588 twin pairs). The columns are as follows: *TwinTrait*= trait number (see Supplementary Table 11), *TraitDescription*=full description of trait,*TaxonomyOfOTU*=taxonomy assignment if trait is OTU, *TraitType*=trait type of either alpha diversity, principal coordinate from beta diversity, OTU, taxonomic group, or collapsed OTU and taxonomic group, *TraitCategory*= trait category of either continuous or categorical.

Supplementary Table 13. Description of European sample traits: Description of European samples continuous traits. The columns are as follows: *EURTrait*= trait number, *TraitDescription*=full description of trait, *TaxonomyOfOTU*=taxonomy assignment if trait is OTU, *TraitCategory*= trait category of either continuous or categorical

Supplementary Table 14. Description of Admixture American sample traits: Description of Admixture American continuous traits. The columns are as follows: *ADMTrait*= trait number, *TraitDescription*=full description of trait, *TaxonomyOfOTU*=taxonomy assignment if trait is OTU, *TraitCategory*= trait category of either continuous or categorical

Supplementary Table 15. Comparison of GCTA and twin model heritability estimates: Comparison of GCTA and Twin Model Heritability Estimates. Comparison. Twin modeling heritability estimates (Standardized A) compared to the "heritability" estimates from GCTA in the European population where "heritability" is defined as the amount of phenotypic variation that is account for by single nucleotide polymorphisms. Comparisons are limited to traits that were categorized as continuous in the twin and European populations (n=40 traits, see Methods, Supplementary Tables 12-13). The columns are as follows: *EURTrait*= European trait coded name (see Supplementary Table 13), GCTA_Heritability =V(G)/Vp (heritability). *GCTA_Heritability_SE* = standard error of V(G)/Vp , *GCTA_Heritability_PVALUE* = P value of V(G)/Vp , *TwinTrait*= twin trait coded name (see Supplementary Table 11), *StandardizedA_Heritability*= standardized A value ("heritability"), *StandardizedALowerCI*= lower 95% confidence interval (CI) bound for Standardized A, *StandardizedAUpperCI*= upper 95% CI for standardized A , *StandardizedAPValue_estimated_from_twinmodel*= P value for Standardized A determined from the twin model, *CorrectedStandardizedAPValue*= corrected P value for standardized A (correcting for 907 tests using the p.adjust package in R (Benjamin&Hochberg method, p.adjust package in R))

Supplementary Table 16. Comparison twin heritability vs. other studies: Comparison of twin heritability estimates in current study (Supplementary Table 5&11) compared to other studies. Comparison of the twin modeling heritability estimates (Standardized A) for the top 44 most heritable traits defined as those traits with a Benjamin-Hochberg corrected P value less

than 1 compared to heritability estimates from other studies (see Supplemental Methods). The columns are as follows: *TwinTrait*= twin trait coded name (see Supplementary Table 11), *Trait Category*= trait category of either continuous or categorical, *TwinModel*=ACE or ADE, *StandardizedA_Heritability*= standardized A value ("heritability"), *StandardizedALowerCI*= lower 95% confidence interval (CI) bound for Standardized A, *StandardizedAUpperCI*= upper 95% CI for standardized A, *StandardizedAPValue_estimated_from_twinmodel*= P value for Standardized A determined from the twin model, *CorrectedStandardizedAPValue*= corrected P value for standardized A (correcting for 907 tests using the p.adjust package in R (Benjamin&Hochberg method, p.adjust package in R)) , *TraitDescription*=full description of trait
